# Supplementary material for: Natural diversity screening, assay development, and characterization of nylon-6 enzymatic depolymerization
Source: Nat Commun. 2024 Feb 9;15:1217. doi: 10.1038/s41467-024-45523-5 (PMC10858056; doi:10.1038/s41467-024-45523-5)
Supplement: Supplementary file 1 — Supplementary Information [file 41467_2024_45523_MOESM1_ESM.pdf]

Supplementary Information

Natural diversity screening, assay development, and characterization of nylon-6 enzymatic depolymerization

Elizabeth L. Bell,[1,2] Gloria Rosetto,[1] Morgan A. Ingraham,[1,2] Kelsey J. Ramirez,[1,2] Clarissa Lincoln,[1,2] Ryan W. Clarke,[1,2] Japheth E. Gado,[1,2] Jacob L. Lilly,[3] Katarzyna H. Kucharzyk,[3] Erika Erickson,[1,2], Gregg T. Beckham[1,2,*]

1. Renewable Resources and Enabling Sciences Center, National Renewable Energy Laboratory, Golden, CO 80401
2. BOTTLE Consortium, Golden, CO 80401
3. Battelle Memorial Institute, Columbus, OH 43201
** Correspondence: gregg.beckham@nrel.gov*

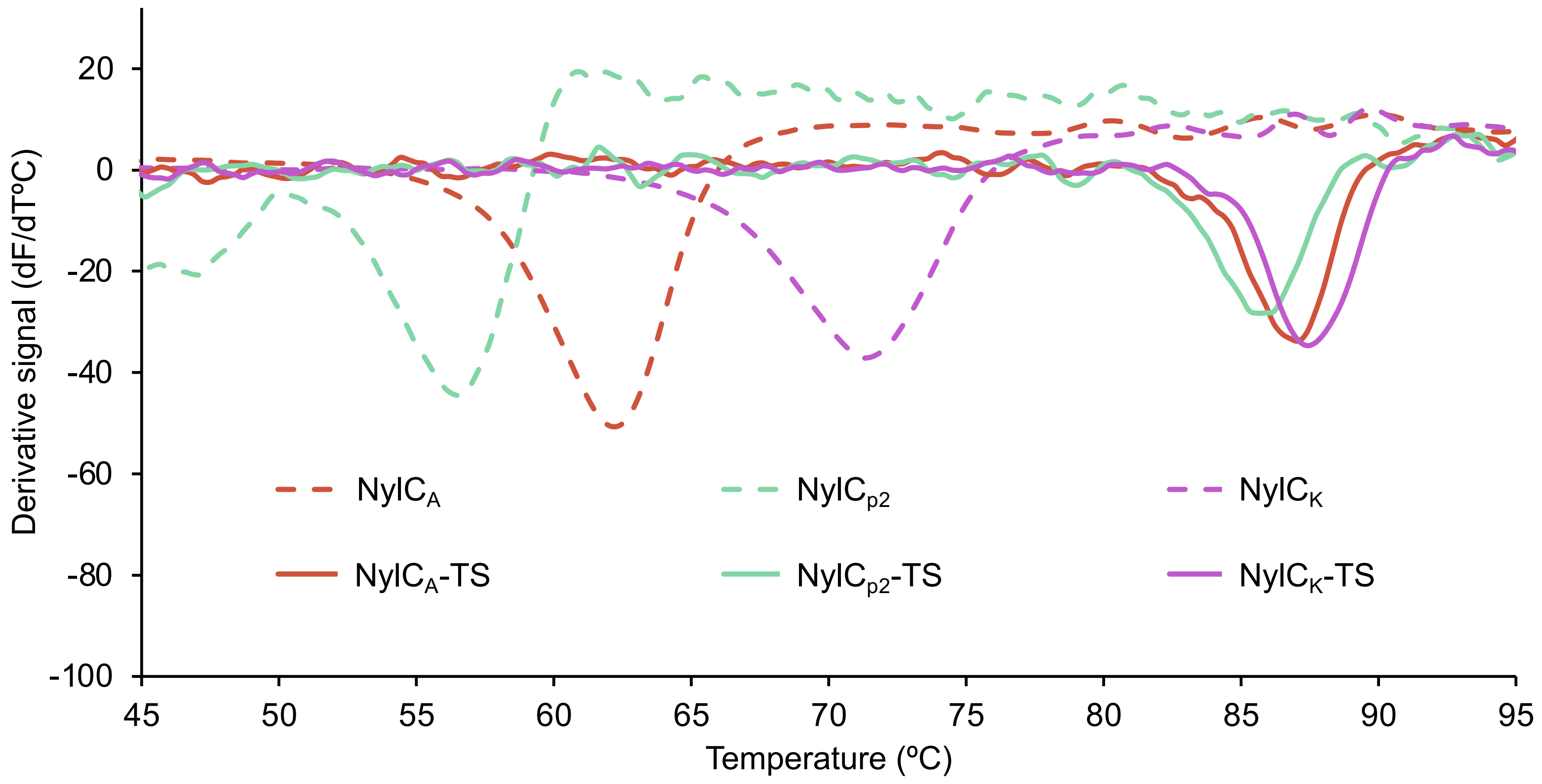

Supplementary Figure 1. Melt curves for wild-type and thermostabilized NylC variants. Melt curve readings were carried out in triplicate (n=3) by differential scanning fluorimetry (DSF). Dashed lines represent wild-type proteins, solid lines represent thermostabilized proteins.

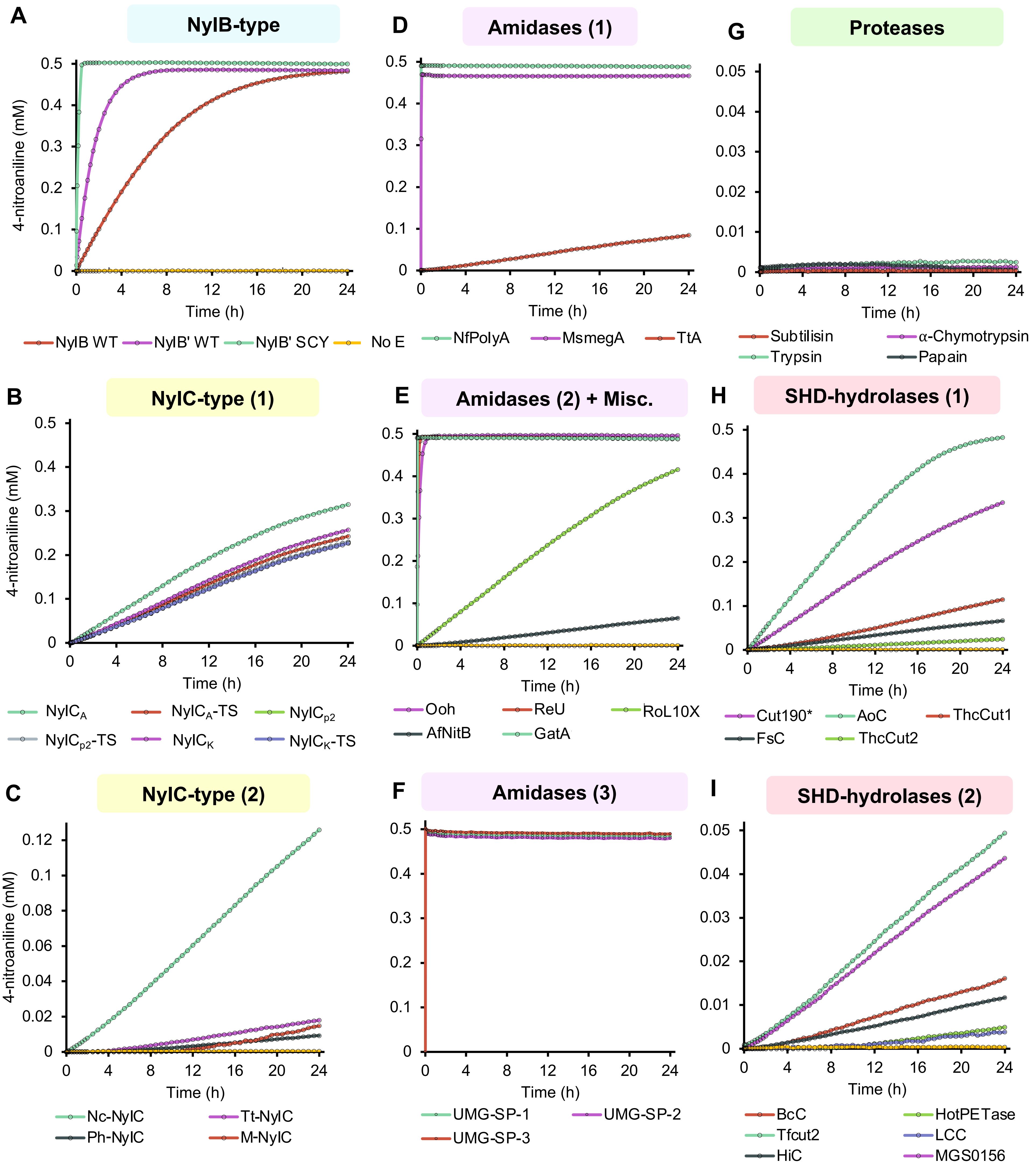

Supplementary Figure 2. Activity assays with *N*-(4-nitrophenyl)butanamide (N4NB) for all enzymes. *p*-NA (*p*-nitroaniline) release profiles of all enzymes tested. Reactions contained 0.5 mM N4NB substrate and 2 µM enzyme in 100 mM sodium phosphate buffer (NaPi), pH 7.5, 150 mM NaCl, and were monitored over the course of 24 h at 30 °C. Enzymes are plotted by grouping: **A.** NylB-type, **B** & **C.** NylC-type, **D, E** & **F.** Amidases and Misc., **G.** Proteases, and **H** & **I.** SHD-hydrolases. Points represent the mean of triplicate measurements (n=3), with the error bars representing the standard deviations of replicate measurements being too small to be visualized. Note that y-axes are shown on different scales to enable different levels of activity to be seen. A no enzyme control reaction (No E) is shown in panel A.

Supplementary Table 1. Materials characterization of standard Goodfellow PA6 film (0.2 mm thickness) before and after enzymatic PA6 film deconstruction reactions. The Goodfellow PA6 film (0.2 mm thickness) analyzed here was used in all reactions unless otherwise specified. Summary of DSC, GPC and TGA data collected throughout the study. The molar mass dispersity index is represented by *Đ*. For the sample preparation, "unmodified" samples were analyzed as received from the supplier or as originally synthesized, apart from DSC measurements, where the PA6 was dried at 40 °C in a vacuum oven for 24 h prior to analysis to remove any residual water, "washed" samples were analyzed following the washing protocol described in the Methods prior to reaction, "no enzyme" samples were incubated in reaction buffer (100 mM NaPi buffer, 150 mM NaCl, pH 7.5) for the described amount of time at the stated temperature in the absence of enzyme, and "+NylCK-TS" samples were analyzed following enzymatic reactions for the described amount of time at the stated temperature (1 µM of enzyme, 0.08 mM enzyme/g PA6 film, 0.65% wt PA6 substrate loading, 100 mM pH 7.5 NaPi buffer with 150 mM NaCl). Dashes represent where no data was collected for that condition set. The mass loss measured during TGA analysis can be attributed to water loss as it occurred at around 100 °C. *All Goodfellow PA6 film used in these analyses came from one batch, as there were small differences between samples of material received from Goodfellow (Supplementary Table 4). Values represent a single measurement (n=1).

Substrate	Sample preparation	Time incubated (days)	T_g (°C)	% Crystallinity	M_n (kDa)	M_w (kDa)	*Đ*	T_D, 50 (°C)	Mass loss (wt%)
Goodfellow PA6 film	Unmodified*	0	50.7	13.3	33.9	50.4	1.5	473.0	0.8
	Washed	0	50.7	14.0	38.4	50.7	1.3	474.5	0.8
Crystalline PA6 film	Unmodified	0	50.6	21.4	28.9	44.4	1.5	472.0	2.0
	Washed	0	49.6	24.0	29.3	44.5	1.5	473.8	2.1
Goodfellow PA6 film	No enzyme (40 °C)	3	47.2	14.2	-	-	-	-	-
		7	57.9	14.2	-	-	-	-	-
		10	46.3	13.7	38.1	50.0	1.3	473.4	1.8
Goodfellow PA6 film	No enzyme (50 °C)	3	46.1	13.7	-	-	-	-	-
		7	47.0	14.9	-	-	-	-	-
		10	48.9	14.8	36.0	48.3	1.3	473.8	1.9
Goodfellow PA6 film	No enzyme (60 °C)	3	42.0	13.4	-	-	-	-	-
		7	48.3	14.0	-	-	-	-	-
		10	46.9	13.5	36.4	47.9	1.3	474.0	2.0
Goodfellow PA6 film	No enzyme (70 °C)	3	56.4	14.6	-	-	-	-	-
		7	49.0	15.0	-	-	-	-	-
		10	56.1	17.2	30.1	41.3	1.4	472.0	1.8
Crystalline PA6 film	No enzyme (60 °C)	10	49.9	21.4	25.0	39.1	1.6	473.8	1.9
Goodfellow PA6 film	+ NylC_K-TS (40 °C)	10	49.8	13.7	35.6	48.2	1.4	474.5	1.6
Goodfellow PA6 film	+ NylC_K-TS (50 °C)	10	47.8	13.8	35.1	47.1	1.3	473.2	1.7
Goodfellow PA6 film	+ NylC_K-TS (60 °C)	10	50.2	12.8	37.9	50.1	1.3	473.8	2.0
Goodfellow PA6 film	+ NylC_K-TS (70 °C)	10	47.6	17.4	36.7	48.5	1.3	470.6	1.6
Crystalline PA6 film	+ NylC_K-TS (60 °C)	10	49.2	21.0	27.6	42.3	1.5	474.2	2.4

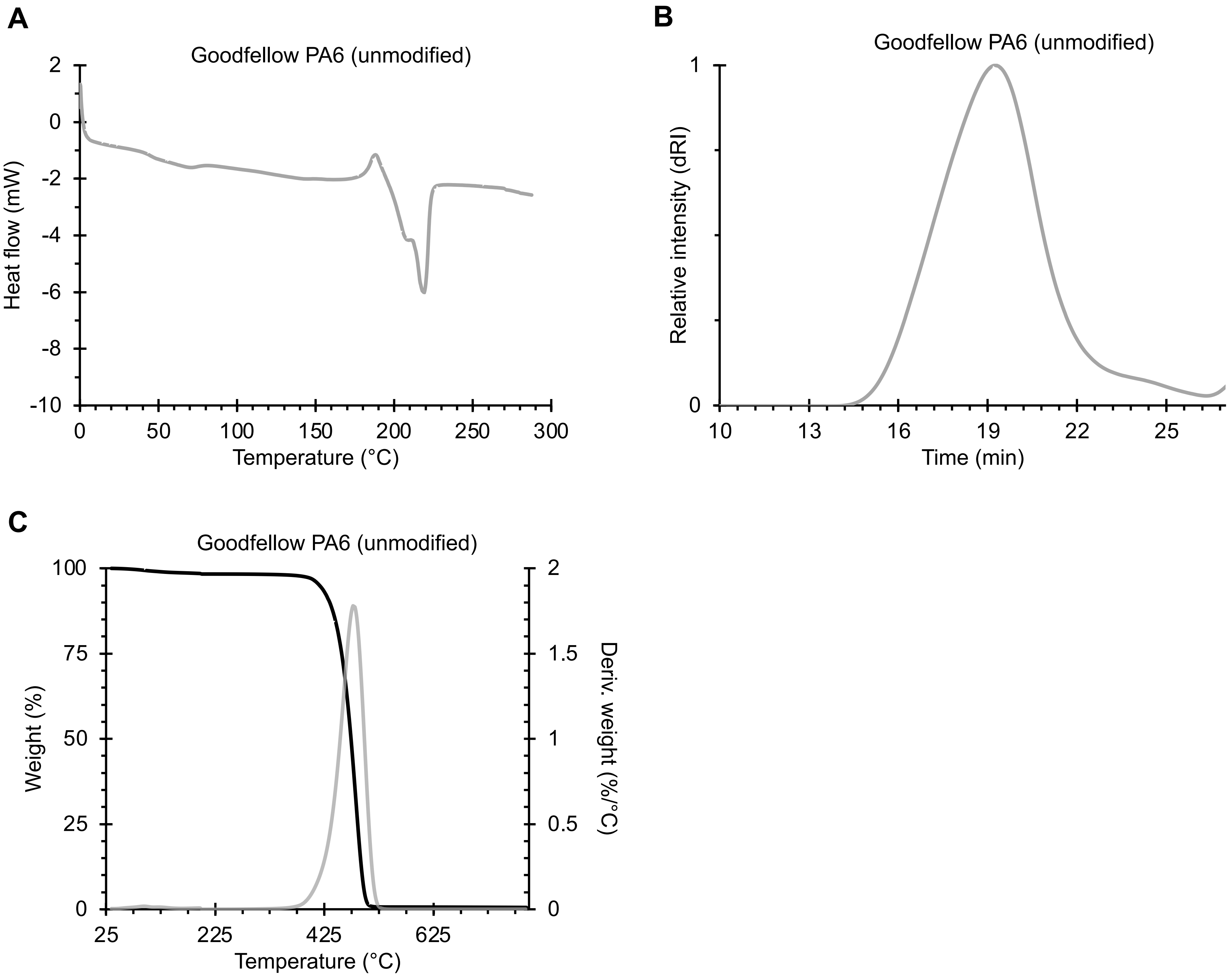

Supplementary Figure 3. Materials characterization plots for Goodfellow PA6 film. The plots are from untreated PA6 film (0.2 mm thickness) as received from Goodfellow (unmodified). **A.** Differential scanning calorimetry (DSC) plot. For DSC, the PA6 was dried at 40 °C for 24 h prior to analysis to remove any residual water. **B.** Gel permeation chromatography (GPC) plot measured with the differential refractive index (dRI) detector. **C.** Thermogravimetric (TGA) plot. For the TGA plot, the darker colored lines represent the weight (%) (left y-axis), and the lighter lines the derivative weight (%) (right y-axis). Plots represent single measurements (n=1).

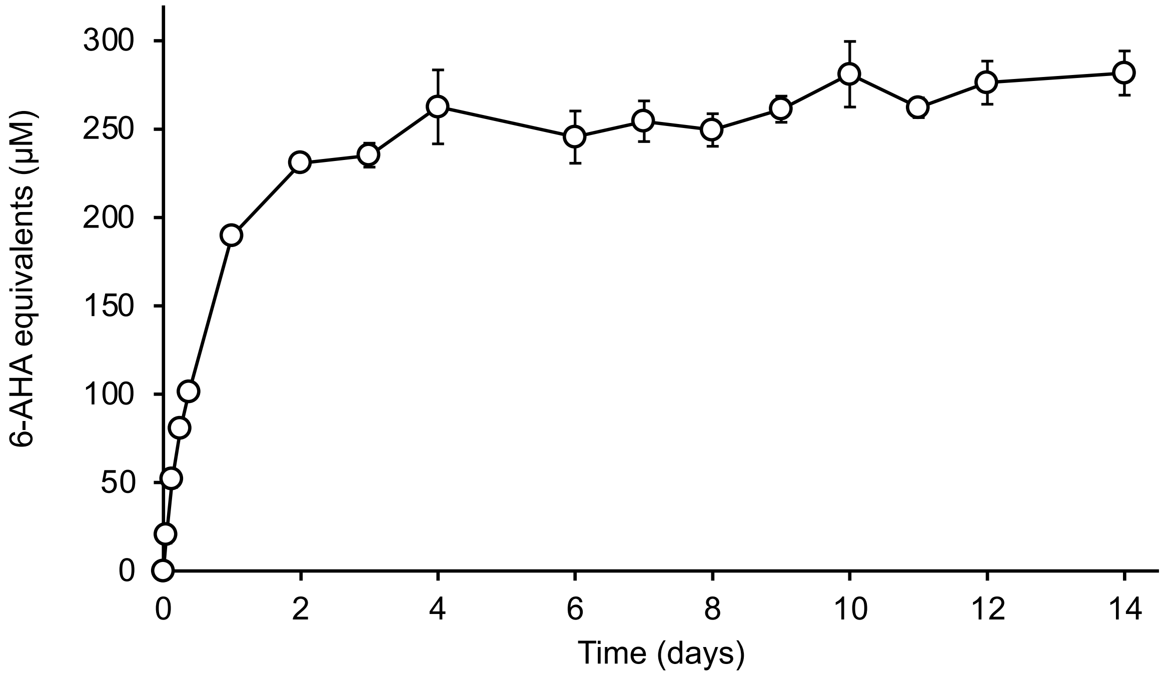

Supplementary Figure 4. Reaction profile of enzymatic PA6 deconstruction. Total released linear 6-AHA oligomers, represented as 6-AHA monomer equivalents, in reactions with 2 µM NylC$_K$-TS and 13 mg PA6 (0.15 mM enzyme/g PA6 film, 0.65 wt% substrate loading) at 60 °C over the course of 14 days, in reaction buffer (100 mM NaPi buffer, pH 7.5, 150 mM NaCl). Reactions were carried out in triplicate (n=3); error bars represent the standard deviation of the replicate measurements; the error bar centers are the means of the replicate measurements; circles represent the mean value of the triplicate measurements.

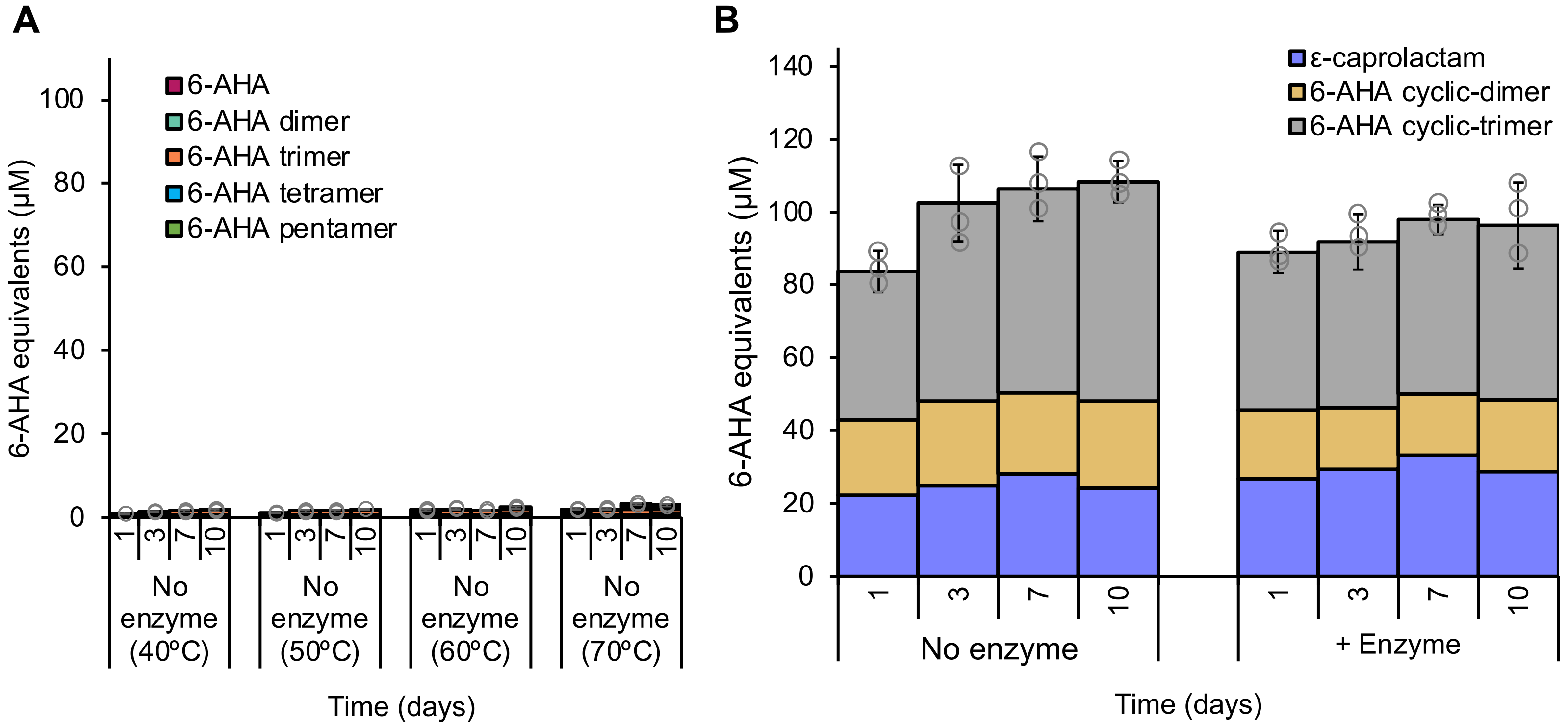

Supplementary Figure 5. Release of PA6 oligomers during reaction. A. There was minimal release of linear oligomers of 6-AHA following incubation of PA6 film (13 mg, 0.65 wt% substrate loading) in reaction buffer (100 mM NaPi, pH 7.5, 150 mM NaCl) over the course of 10 days from 40-70 °C. **B.** Total released cyclic 6-AHA oligomers following reaction with enzyme (+ Enzyme, 2 µM LCC-ICCG) and 13 mg PA6 (0.15 mM enzyme/g PA6 film, 0.65 wt% substrate loading) or without enzyme (no enzyme) over the course of 10 days (60 °C, 100 mM NaPi buffer, pH 7.5, 150 mM NaCl). Cyclic-oligomer concentrations shown here are representative of all enzymes apart from NylC-type, GatA and UMG-SP-1. For both charts, reactions were carried out in triplicate (n=3), error bars show the standard deviation of the replicate measurements, the error bar centers are the means of the replicate measurements, and the replicate measurements are represented as grey circles.

Supplementary Table 2. Stability of linear PA6 oligomer standards following incubation. Chemical standards of 6-AHA monomer, 6-AHA dimer and 6-AHA trimer (10 µg mL^{-1}) were individually incubated over the course of 10 days from 40-80 °C in reaction buffer (100 mM NaPi buffer, pH 7.5, 150 mM NaCl). The values represent the percentage recovery of a 10 µg mL^{-1} standard for each compound following incubation at the stated temperature and duration, as measured by LC-MS/MS. Reactions were conducted in triplicate (n=3), with the S.D. representing the standard deviation of the replicate measurements.

**6-AHA monomer**			
Temperature	Day	Percentage recovery (%)	S.D.
40 °C	1	101.4	0.9
	3	103.6	1.6
	7	94.3	1.2
	10	93.3	1.1
50 °C	1	99.8	0.3
	3	101.9	1.7
	7	101.0	1.5
	10	87.8	2.9
60 °C	1	100.1	4.8
	3	103.1	4.1
	7	93.9	0.2
	10	75.7	2.0
70 °C	1	99.7	1.2
	3	109.0	2.2
	7	100.6	2.5
	10	90.0	2.9
80 °C	1	103.8	6.7
	3	101.7	0.9
	7	95.0	3.7
	10	72.3	7.4

**6-AHA dimer**			
Temperature	Day	Percentage recovery (%)	S.D.
40 °C	1	105.4	0.3
	3	109.0	1.6
	7	94.6	1.5
	10	85.5	1.6
50 °C	1	104.3	2.0
	3	101.5	0.1
	7	109.1	1.0
	10	89.5	0.9
60 °C	1	102.7	0.1
	3	106.1	4.8
	7	97.0	6.5
	10	97.2	0.8
70 °C	1	102.1	1.6
	3	101.4	1.0
	7	97.1	0.8
	10	104.1	1.0
80 °C	1	97.4	5.2
	3	105.3	0.7
	7	95.4	0.3
	10	98.0	3.1

**6-AHA trimer**			
Temperature	Day	Percentage recovery (%)	S.D.
40 °C	1	103.5	7.2
	3	115.1	5.0
	7	102.8	0.7
	10	96.7	2.6
50 °C	1	101.3	3.9
	3	107.5	0.6
	7	114.5	0.9
	10	95.7	1.6
60 °C	1	105.1	0.7
	3	107.1	2.2
	7	105.4	2.0
	10	106.3	1.1
70 °C	1	113.0	0.2
	3	109.6	7.1
	7	103.1	1.9
	10	99.9	0.9
80 °C	1	94.7	3.8
	3	113.7	1.9
	7	103.8	3.7
	10	87.0	5.3

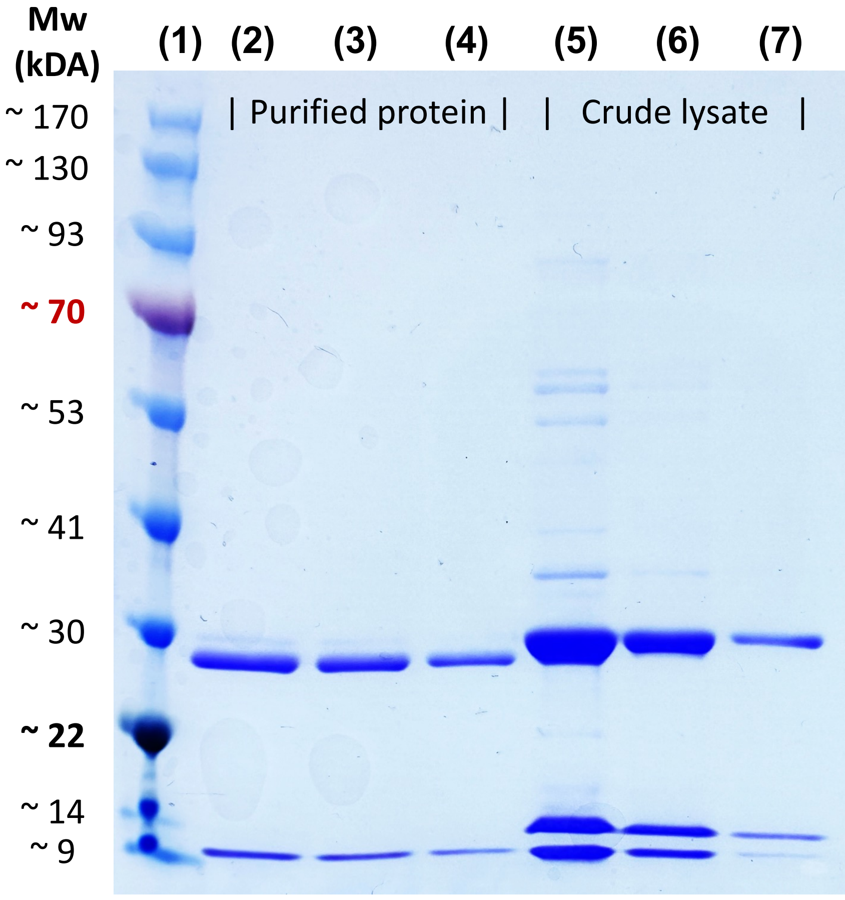

Supplementary Figure 6. SDS-PAGE gel of purified NylC$_K$-TS and NylC$_K$-TS crude cell lysate. NylC$_K$-TS was prepared as either a purified protein or crude cell lysate as detailed in the Methods. Lanes contain the following samples (1) ladder, (2) 1 mg mL^{-1} NylC$_K$-TS, (3) 0.5 mg mL^{-1} NylC$_K$-TS, (4) 0.1 mg mL^{-1} NylCK-TS, (5) 100% crude cell lysate, (6) 50% crude cell lysate, (7) 10% crude cell lysate. Purified NylC$_K$-TS appears as two bands on the gel (~9 kDa and ~27 kDA), as its component subunits dissociate. The major contaminant band in the crude cell lysate samples is assumed to be lysozyme (~15 kDA) from the chemical lysis preparation. The gel was prepared twice, with the gel shown being representative of the set. The uncropped gel is available in Supplementary Figure 32.

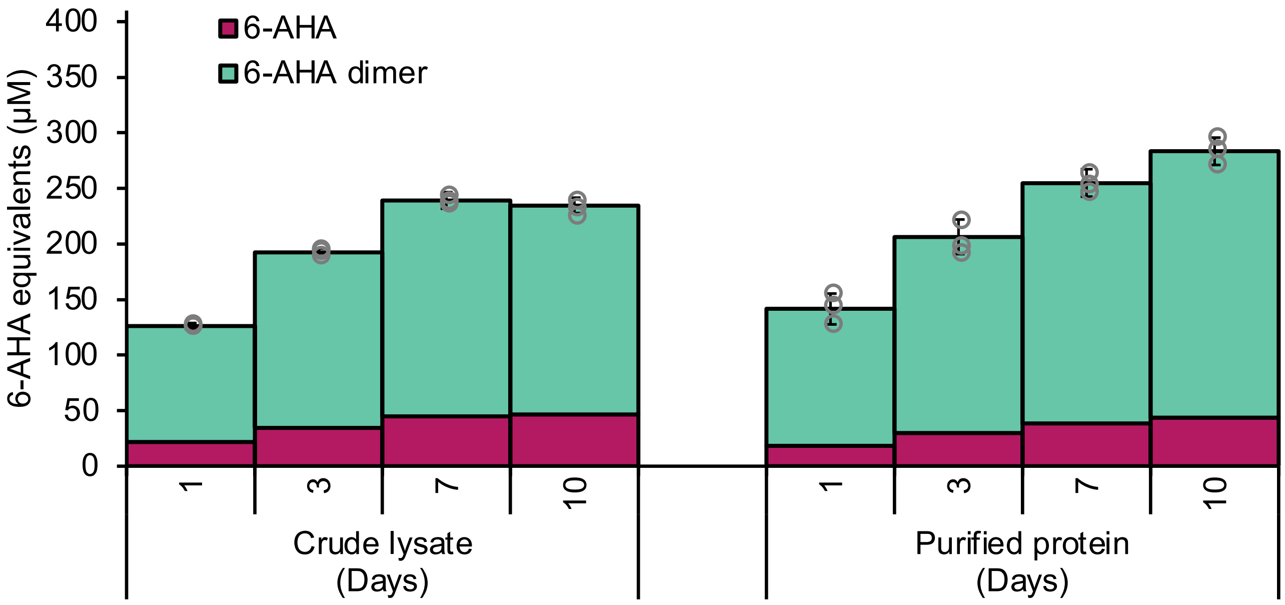

Supplementary Figure 7. Reaction profile of enzymatic PA6 deconstruction with crude cell lysate or purified protein. Total released linear 6-AHA oligomers, represented as 6-AHA monomer equivalents, in reactions with with either 100 µL of NylC$_K$-TS containing cell lysate (Crude lysate) as the biocatalytic agent, or 1 µM purified NylC$_K$-TS (Purified protein) with 13 mg PA6 (0.08 mM enzyme/g PA6 film in the purified enzyme reaction, 0.65 wt% substrate loading) at 60 °C over the course of 10 days, in reaction buffer (100 mM NaPi buffer, pH 7.5, 150 mM NaCl). Reactions were carried out in triplicate (n=3); error bars represent the standard deviation of the replicate measurements; the error bar centers are the means of the replicate measurements; the replicate measurements are represented as grey circles.

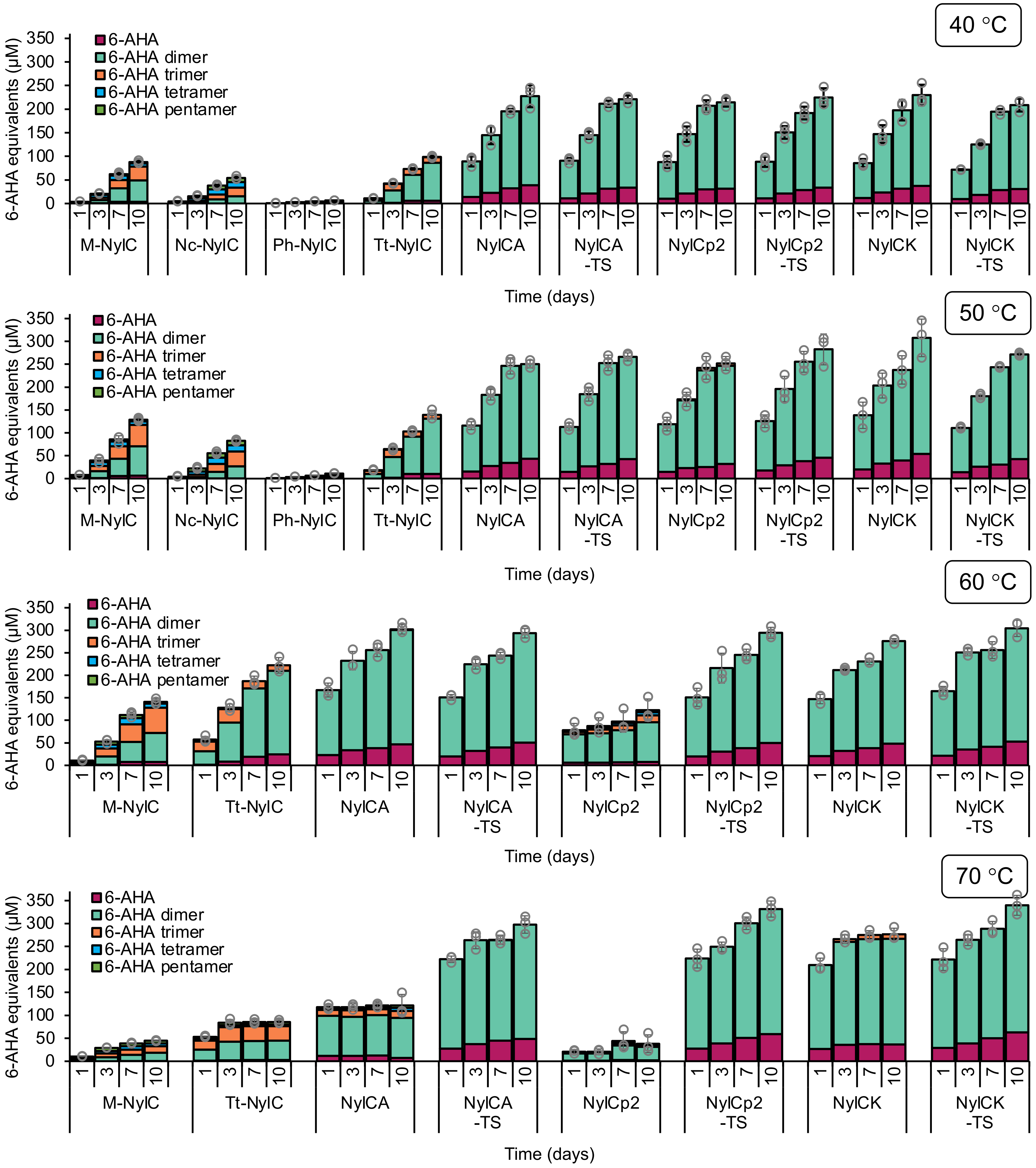

Supplementary Figure 8. Reactions of NylC-type enzymes with PA6. Total released linear 6-AHA oligomers from enzymes in the NylC-type group, following reaction with PA6 film. Reactions contained 2 μM enzyme and 13 mg PA6 (0.15 mM enzyme/g PA6 film, 0.65 wt% substrate loading) and were incubated from 40-70 °C over the course of 10 days in reaction buffer (100 mM NaPi buffer, pH 7.5, 150 mM NaCl). Above 50 °C, reactions with enzymes that led to no detectable product release above background are not shown. Reactions were carried out in triplicate (n=3), error bars show the standard deviation of the replicate measurements, the error bar centers are the means of the replicate measurements, and the replicate measurements are represented as grey circles.

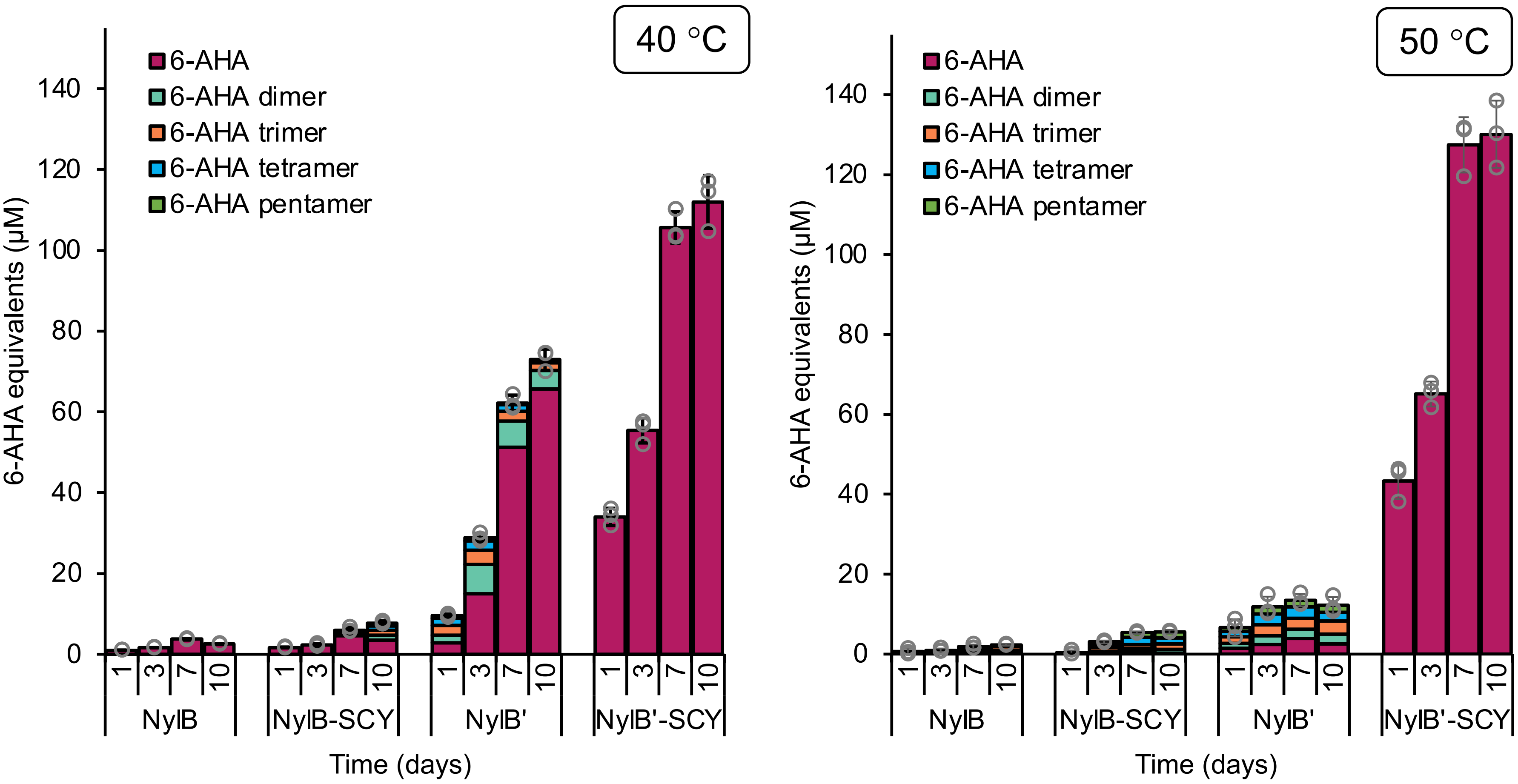

Supplementary Figure 9. Reactions of NylB-type enzymes with PA6. Total released linear 6-AHA oligomers from enzymes in the NylB-type group, following reaction with PA6 film. Reactions contained 2 µM enzyme and 13 mg PA6 (0.15 mM enzyme/g PA6 film, 0.65 wt% substrate loading) and were incubated from 40-70 °C over the course of 10 days in reaction buffer (100 mM NaPi buffer, pH 7.5, 150 mM NaCl). Above 50 °C, minimal product release above background was seen with any of the NylB-type enzymes. Reactions were carried out in triplicate (n=3), error bars show the standard deviation of the replicate measurements, the error bar centers are the means of the replicate measurements, and the replicate measurements are represented as grey circles.

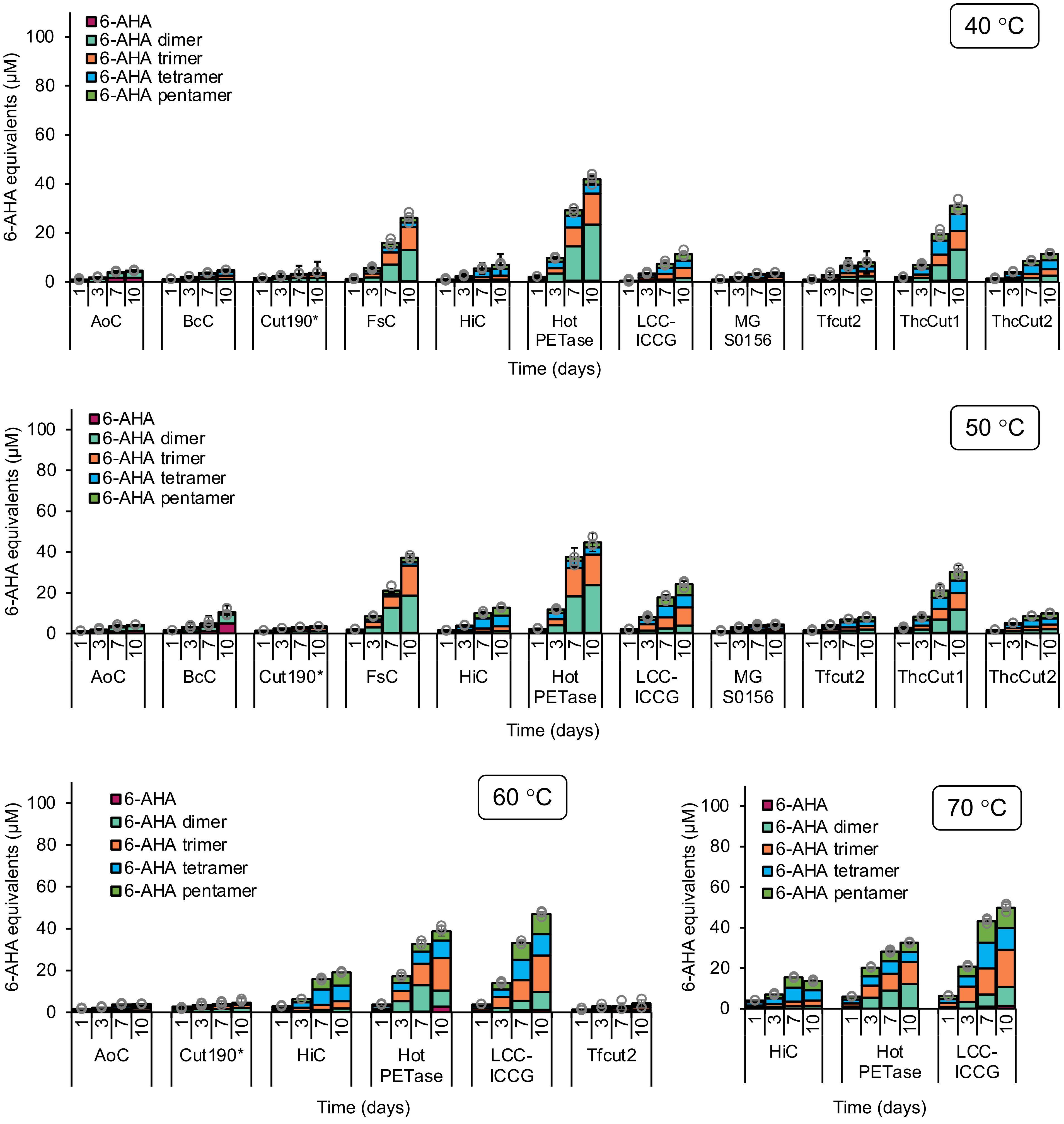

Supplementary Figure 10. Reactions of SHD-hydrolases with PA6. Total released linear 6-AHA oligomers with enzymes in the SHD-hydrolase group following reaction with PA6 film. Reactions contained 2 µM enzyme and 13 mg PA6 (0.15 mM enzyme/g PA6 film, 0.65 wt% substrate loading) and were incubated from 40-70 °C over the course of 10 days in reaction buffer (100 mM NaPi buffer, pH 7.5, 150 mM NaCl). Above 50 °C, reactions with enzymes that led to no detectable product release above background are not shown. Reactions were carried out in triplicate (n=3), error bars show the standard deviation of the replicate measurements, the error bar centers are the means of the replicate measurements, and the replicate measurements are represented as grey circles.

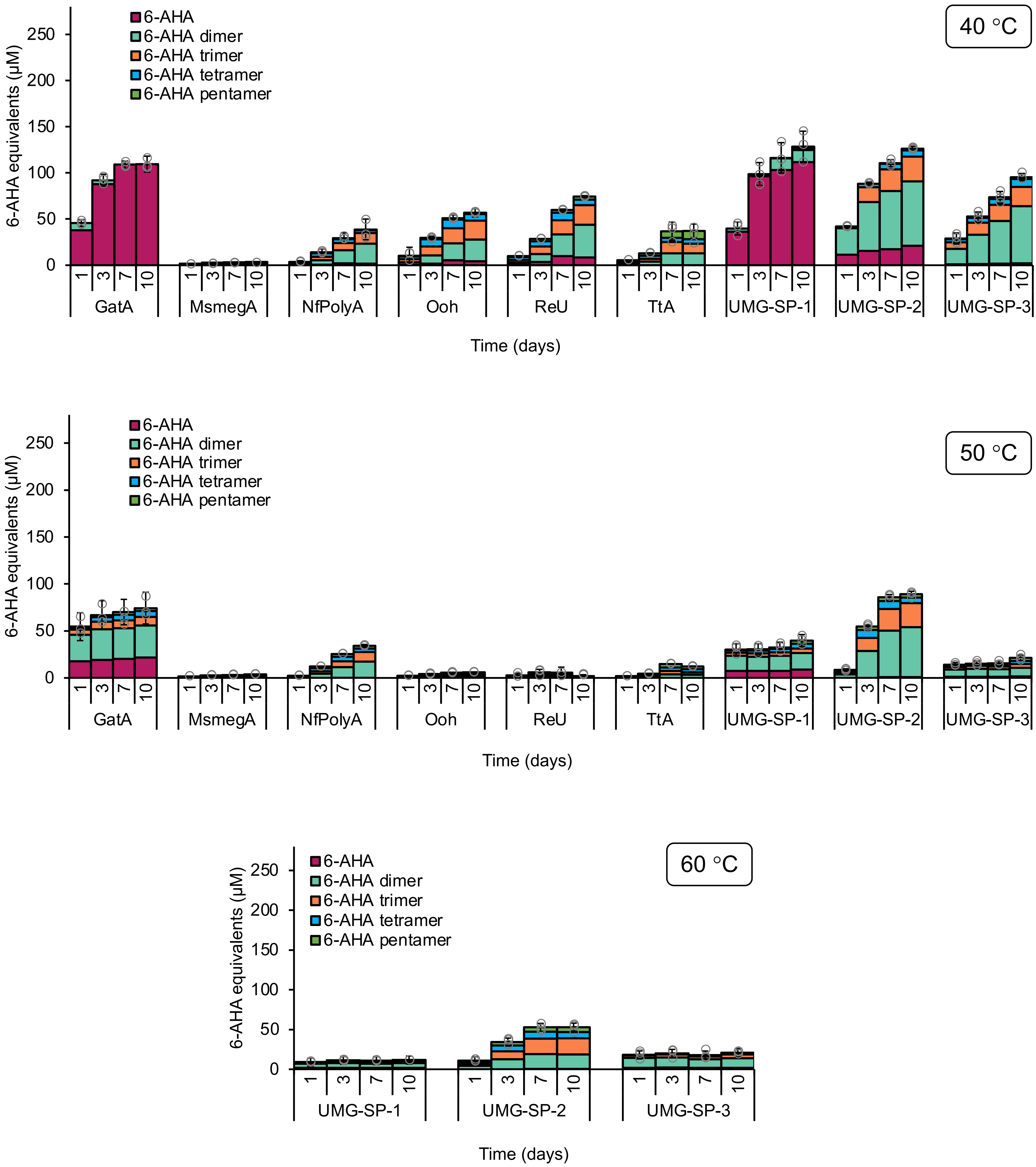

Supplementary Figure 11. Reactions of amidases with PA6. Total released linear 6-AHA oligomers with enzymes in the amidase group following reaction with PA6 film. Reactions contained 2 μM enzyme and 13 mg PA6 (0.15 mM enzyme/g PA6 film, 0.65 wt% substrate loading) and were incubated from 40-70 °C over the course of 10 days in reaction buffer (100 mM NaPi buffer, pH 7.5, 150 mM NaCl). Above 50 °C, reactions with enzymes that led to no detectable product release above background are not shown. Reactions were carried out in triplicate (n=3), error bars show the standard deviation of the replicate measurements, the error bar centers are the means of the replicate measurements, and the replicate measurements are represented as grey circles.

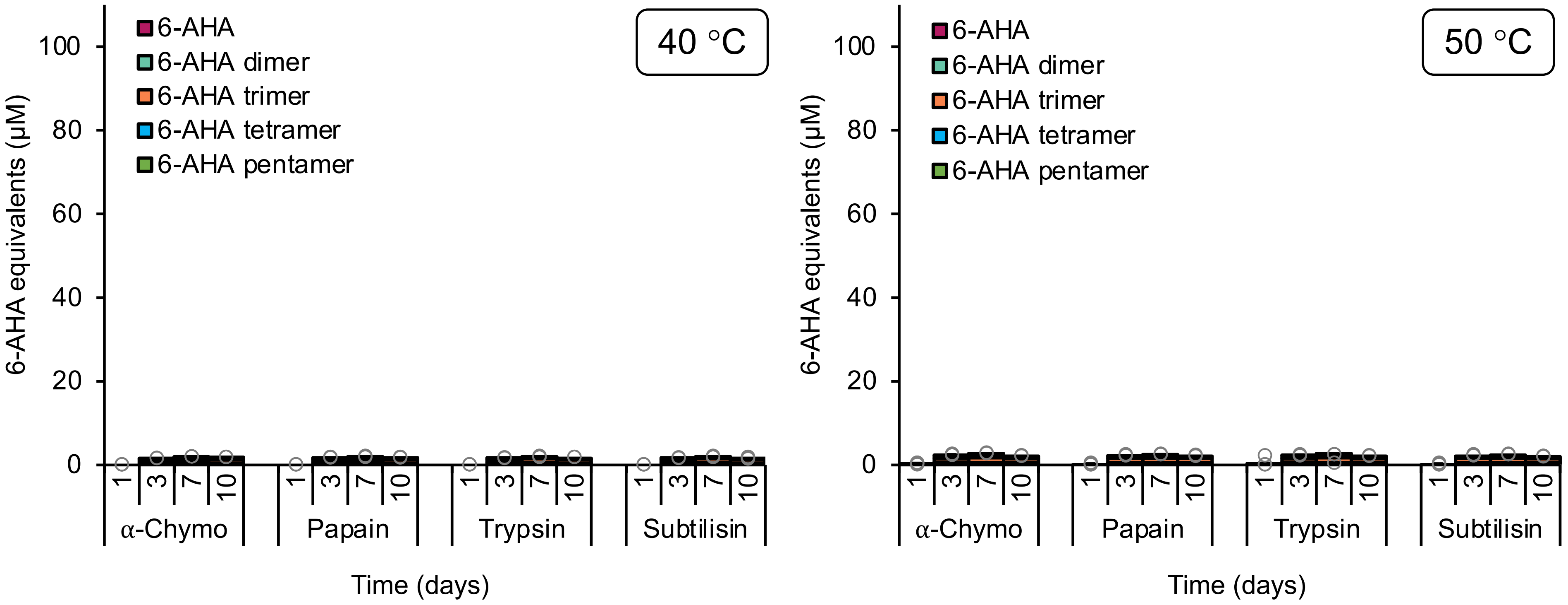

Supplementary Figure 12. Reactions of proteases with PA6. Total released linear 6-AHA oligomers with enzymes in the protease group, following reaction with PA6 film. Reactions contained 2 µM enzyme and 13 mg PA6 (0.15 mM enzyme/g PA6 film, 0.65 wt% substrate loading) and were incubated from 40-70 °C over the course of 10 days in reaction buffer (100 mM NaPi buffer, pH 7.5, 150 mM NaCl). Minimal product release above background levels was seen at any reaction condition, with representative reactions at 40-50 °C shown. Reactions were carried out in triplicate (n=3), error bars show the standard deviation of the replicate measurements, the error bar centers are the means of the replicate measurements, and the replicate measurements are represented as grey circles.

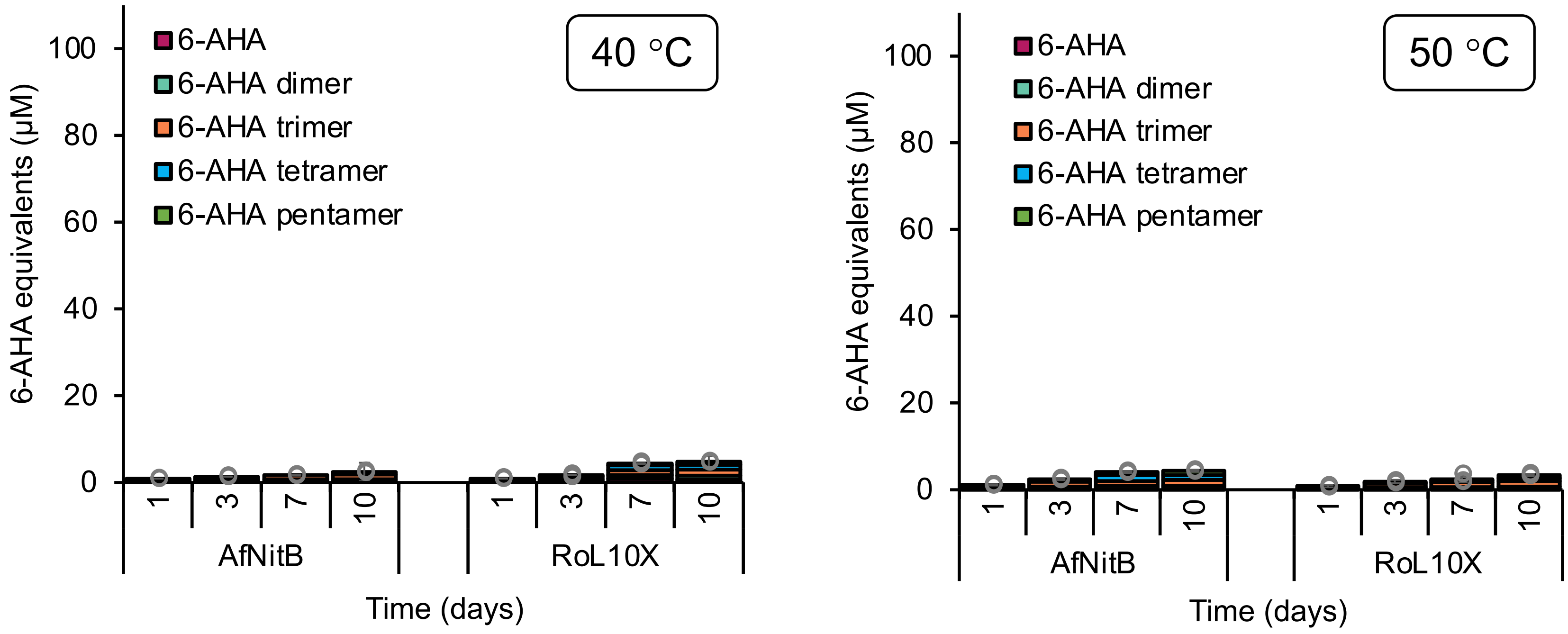

Supplementary Figure 13. Reactions of AfNitB and RoL10X with PA6. Total released linear 6-AHA oligomers with enzymes not in one of the five main groups (misc.), following reaction with PA6 film. Reactions contained 2 µM enzyme and 13 mg PA6 (0.15 mM enzyme/g PA6 film, 0.65 wt% substrate loading) and were incubated from 40-70 °C over the course of 10 days in reaction buffer (100 mM NaPi buffer, pH 7.5, 150 mM NaCl). Minimal additional product release above background levels was seen under any reaction condition, with representative reactions at 40-50 °C shown. Reactions were carried out in triplicate (n=3), error bars show the standard deviation of the replicate measurements, the error bar centers are the means of the replicate measurements, and the replicate measurements are represented as grey circles.

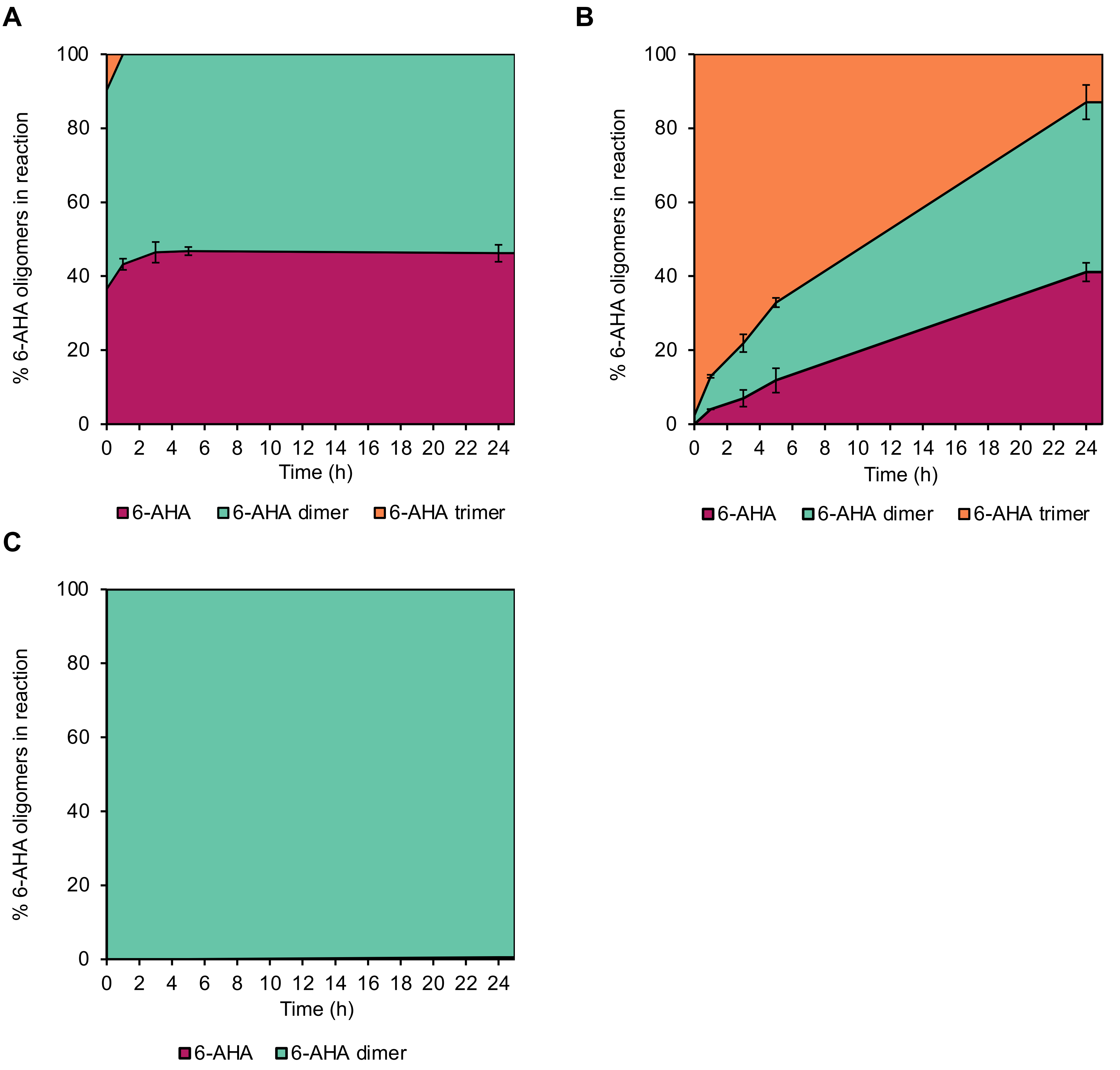

Supplementary Figure 14. Reactions of NylC$_K$-TS and Tt-NylC with 6-AHA linear oligomers. A. Reaction of NylC$_K$-TS with 6-AHA linear trimer for 24 h at reaction conditions (50 µM 6-AHA trimer, pH 7.5 NaPi buffer, 150 mM NaCl, 2 µM enzyme, 60 °C), showing the change in 6-AHA oligomers as a percentage of total oligomers in the reaction. **B.** Reaction of Tt-NylC with 6-AHA linear trimer for 24 h at reaction conditions (50 µM 6-AHA trimer, pH 7.5 NaPi buffer, 150 mM NaCl, 2 µM enzyme, 60 °C), showing the change in 6-AHA oligomers as a percentage of total oligomers in the reaction. **C.** Reaction of NylC$_K$-TS with 6-AHA linear dimer for 24 h at reaction conditions (100 µM 6-AHA dimer, pH 7.5 NaPi buffer, 150 mM NaCl, 2 µM enzyme, 60 °C), showing the change in 6-AHA oligomers as a percentage of total oligomers in the reaction. For both 6-AHA dimer and 6-AHA trimer, there was no change in concentration in no enzyme control reactions. For A, B, and C, reactions were carried out in triplicate (n=3); error bars represent the standard deviation of the replicate measurements, and the error bar centers are the means of the replicate measurements.

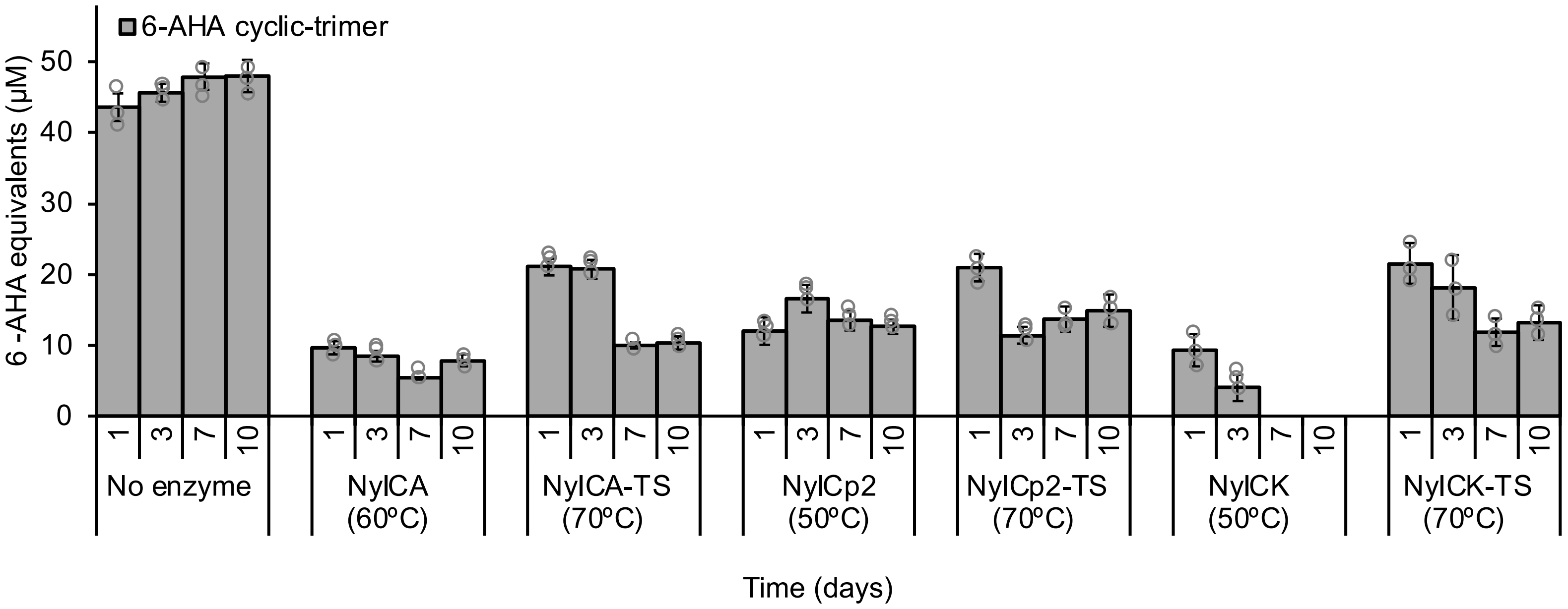

Supplementary Figure 15. Concentration of 6-AHA cyclic-trimer in reactions with the most active NylCs. Concentrations of 6-AHA cyclic-trimer following reactions of NylC-type enzymes with PA6 film versus a no enzyme control. For enzymatic reactions, reactions contained 2 μM enzyme and 13 mg PA6 (0.15 mM enzyme/g PA6 film, 0.65 wt% substrate loading) and were incubated at the enzyme's optimal temperature (x-axis) over the course of 10 days in reaction buffer (100 mM NaPi buffer, pH 7.5, 150 mM NaCl). No enzyme reactions were treated in the same way, but without enzyme addition. Reactions were carried out in triplicate (n=3), error bars show the standard deviation of the replicate measurements, the error bar centers are the means of the replicate measurements, and the replicate measurements are represented as grey circles.

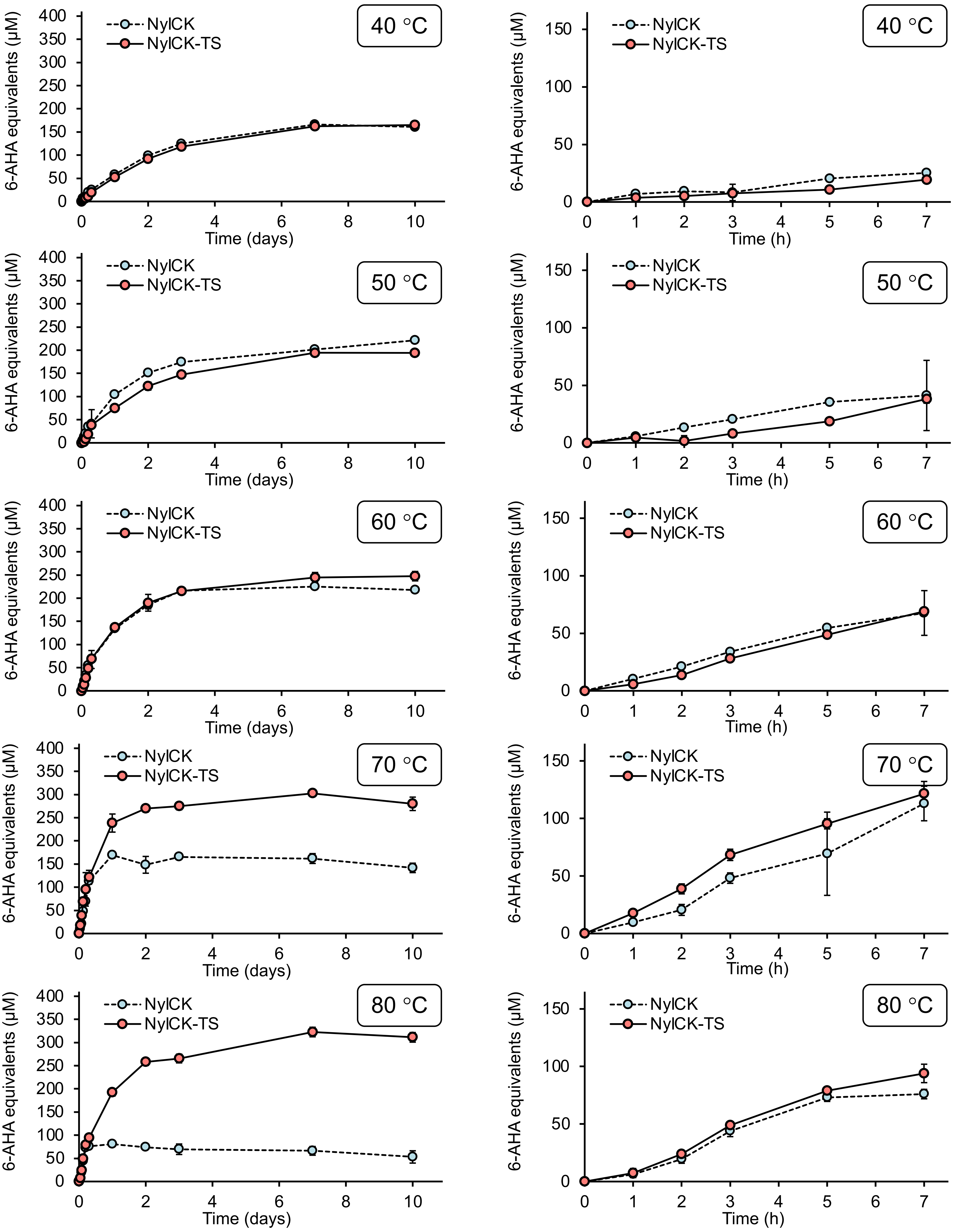

Supplementary Figure 16. Comparative reactions of NylC$_K$ and NylC$_K$-TS. Total released linear 6-AHA oligomers with NylC$_K$ and NylC$_K$-TS. Reactions contained 1 µM enzyme and 13 mg PA6 (0.08 mM enzyme/g PA6 film, 0.65 wt% substrate loading) and were incubated from 40-80 °C over the course of 10 days in reaction buffer (100 mM NaPi buffer, pH 7.5, 150 mM NaCl). Graphs to the right present a zoom in of the first seven hours of reaction. Reactions were carried out in triplicate (n=3), error bars show the standard deviation of the replicate measurements, and the error bar centers are the means of the replicate measurements.

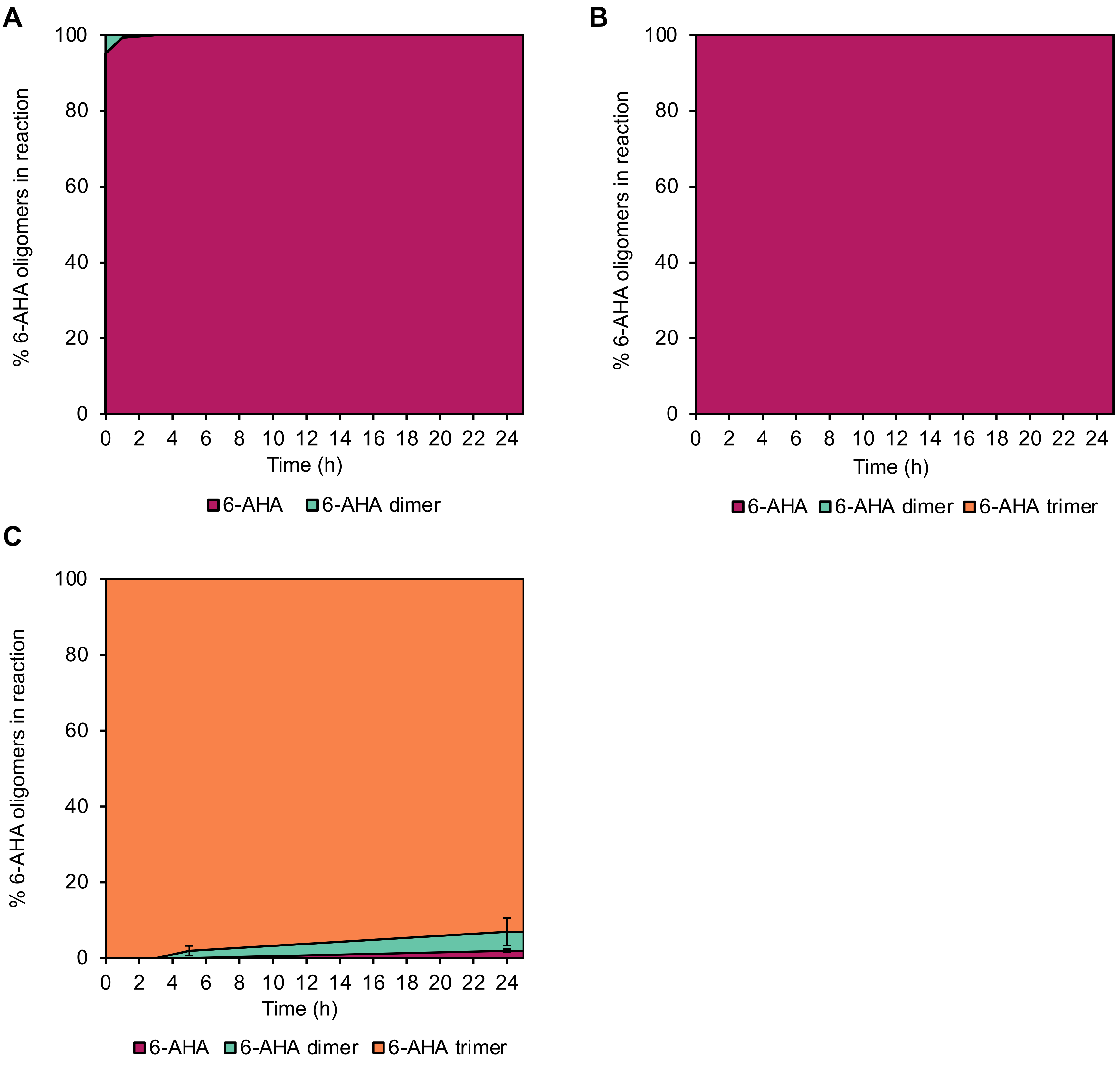

Supplementary Figure 17. Reactions of NylB'-SCY and LCC-ICCG with 6-AHA linear oligomers. A. Reaction of NylB'-SCY with 6-AHA linear dimer for 24 h at reaction conditions (100 µM 6-AHA dimer, pH 7.5 NaPi buffer, 150 mM NaCl, 2 µM enzyme, 70 °C), showing the change in 6-AHA oligomers as a percentage of total oligomers in the reaction. **B.** Reaction of NylB'-SCY with 6-AHA linear trimer for 24 h at reaction conditions (50 µM 6-AHA trimer, pH 7.5 NaPi buffer, 150 mM NaCl, 2 µM enzyme, 70 °C), showing the change in 6-AHA oligomers as a percentage of total oligomers in the reaction. **C.** Reaction of LCC-ICCG with 6-AHA linear trimer for 24 h at reaction conditions (50 µM 6-AHA trimer, pH 7.5 NaPi buffer, 150 mM NaCl, 2 µM enzyme, 70 °C), showing the change in 6-AHA oligomers as a percentage of total oligomers in the reaction. For A, B, and C, reactions were carried out in triplicate (n=3); error bars represent the standard deviation of the replicate measurements; the error bar centers are the means of the replicate measurements.

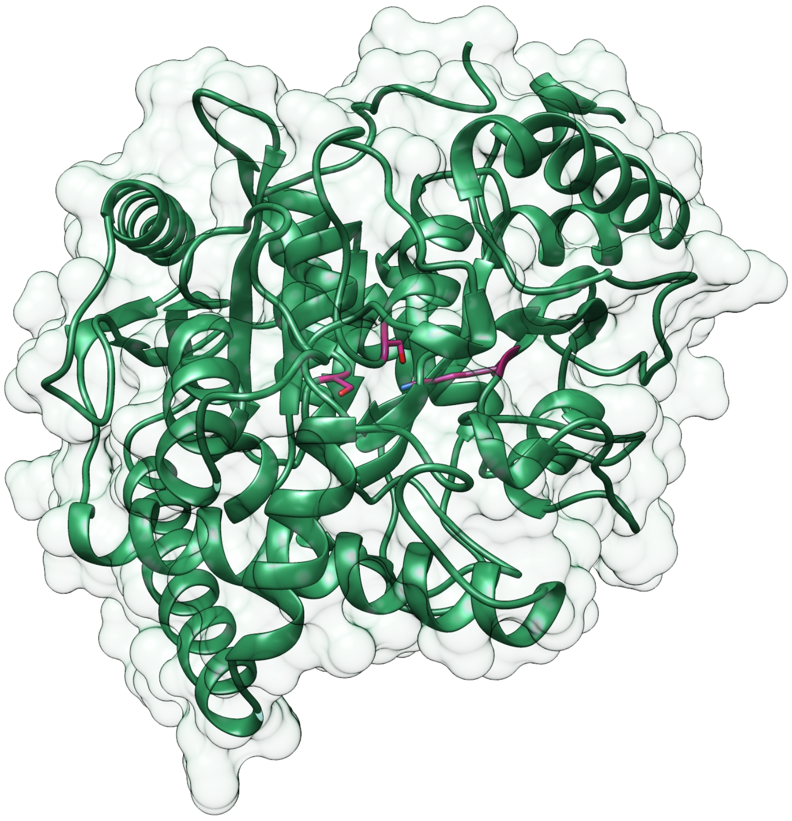

Supplementary Figure 18. AlphaFold predicted structure of NfPolyA. The green ribbon represents the structure of NfPolyA as predicted using AlphaFold. The catalytic triad is shown in the center of the protein, in stick representation colored by all atoms, with pink carbon atoms.

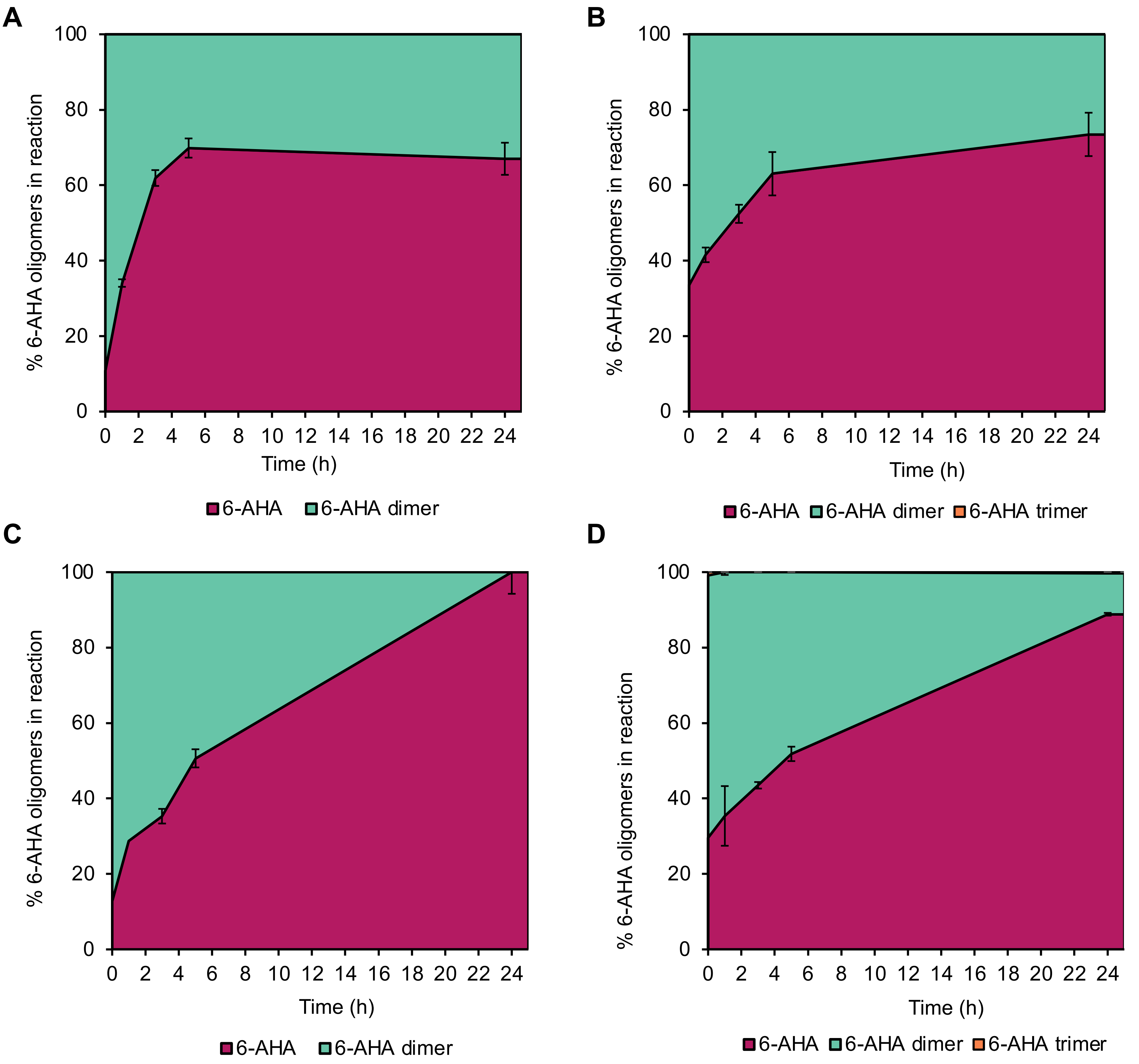

Supplementary Figure 19. Reactions of GatA and UMG-SP-1 with 6-AHA linear oligomers. A. Reaction of GatA with 6-AHA linear dimer for 24 h at reaction conditions (100 µM 6-AHA dimer, pH 7.5 NaPi buffer, 150 mM NaCl, 2 µM enzyme, 40 °C), showing the change in 6-AHA oligomers as a percentage of total oligomers in the reaction. **B.** Reaction of GatA with 6-AHA linear trimer for 24 h at reaction conditions (50 µM 6-AHA trimer, pH 7.5 NaPi buffer, 150 mM NaCl, 2 µM enzyme, 40 °C), showing the change in 6-AHA oligomers as a percentage of total oligomers in the reaction. **C.** Reaction of UMG-SP-1 with 6-AHA linear dimer for 24 h at reaction conditions. (100 µM 6-AHA dimer, pH 7.5 NaPi buffer, 150 mM NaCl, 2 µM enzyme, 40 °C), showing the change in 6-AHA oligomers as a percentage of total oligomers in the reaction. **D.** Reaction of UMG-SP-1 with 6-AHA linear trimer for 24 h at reaction conditions (50 µM 6-AHA trimer, pH 7.5 NaPi buffer, 150 mM NaCl, 2 µM enzyme, 40 °C), showing the change in 6-AHA oligomers as a percentage of total oligomers in the reaction. For both 6-AHA dimer and 6-AHA trimer, there was no change in concentration in no enzyme control reactions. For A, B, C and D, reactions were carried out in triplicate (n=3); error bars represent the standard deviation of the replicate measurements; the error bar centers are the means of the replicate measurements.

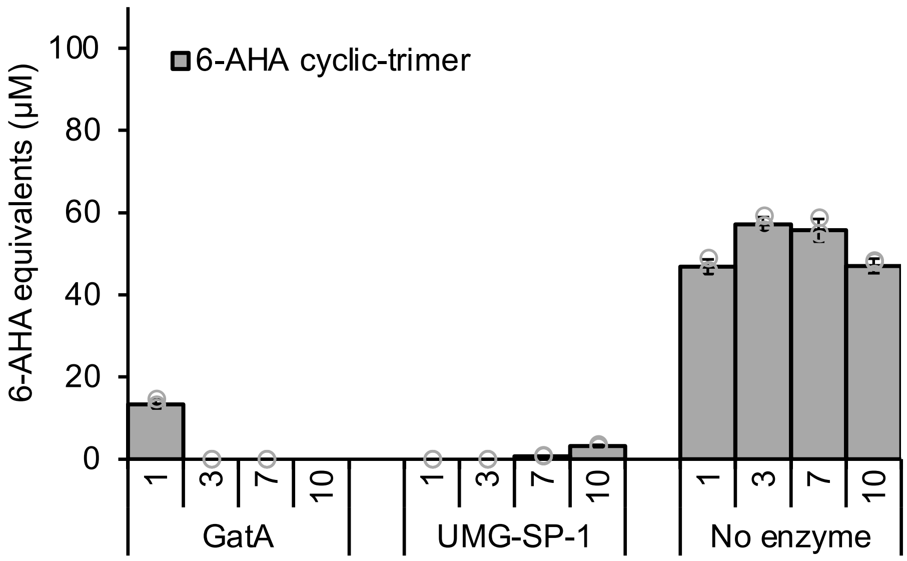

Supplementary Figure 20. Concentration of 6-AHA cyclic-trimer in reactions with GatA and UMG-SP-1. Concentration of 6-AHA cyclic-trimer, following reaction of GatA and UMG-SP-1 with PA6 film, versus a no enzyme control. Enzyme containing reactions contained 2 µM enzyme and 13 mg PA6 (0.15 mM enzyme/g PA6 film, 0.65 wt% substrate loading) and were incubated at 40 °C over the course of 10 days in reaction buffer (100 mM NaPi buffer, pH 7.5, 150 mM NaCl). No enzyme reactions were treated in the same way, but without enzyme addition. Reactions were carried out in triplicate (n=3), error bars show the standard deviation of the replicate measurements, the error bar centers are the means of the replicate measurements, and the replicate measurements are represented as grey circles.

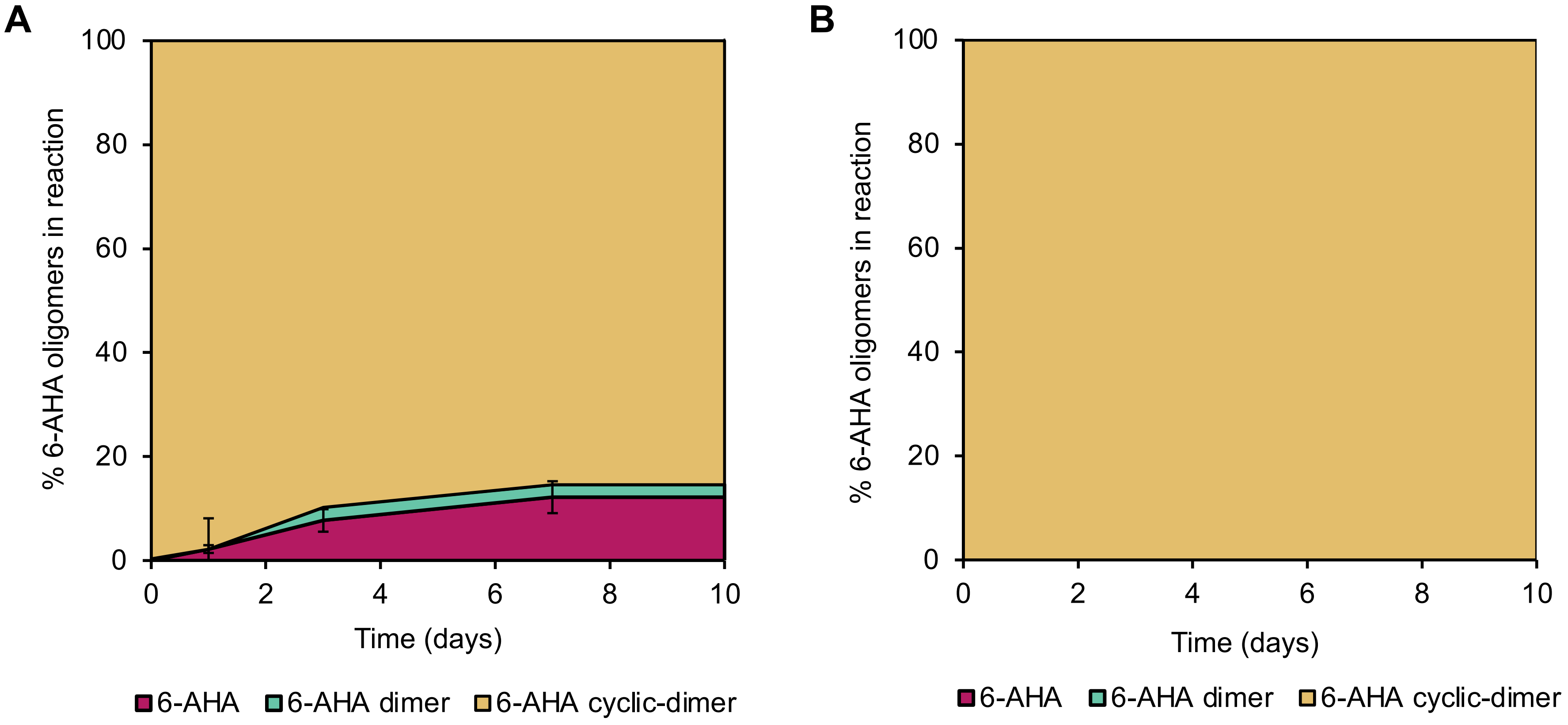

Supplementary Figure 21. Reactions of GatA with 6-AHA cyclic-dimer. A. Reaction of GatA with 6-AHA cyclic-dimer for 24 h at reaction conditions (100 µM 6-AHA cyclic-dimer, pH 7.5 NaPi buffer, 150 mM NaCl, 2 µM enzyme, 40 °C), showing the change in 6-AHA oligomers as a percentage of total oligomers in the reaction. **B.** No enzyme control reaction of 6-AHA cyclic-dimer incubated for 24 h at reaction conditions (100 µM 6-AHA cyclic-dimer, pH 7.5 NaPi buffer, 150 mM NaCl, 40 °C), showing the change in 6-AHA oligomers as a percentage of total oligomers in the reaction. For A and B, reactions were carried out in triplicate (n=3); error bars represent the standard deviation of the replicate measurements, and the error bar centers are the means of the replicate measurements.

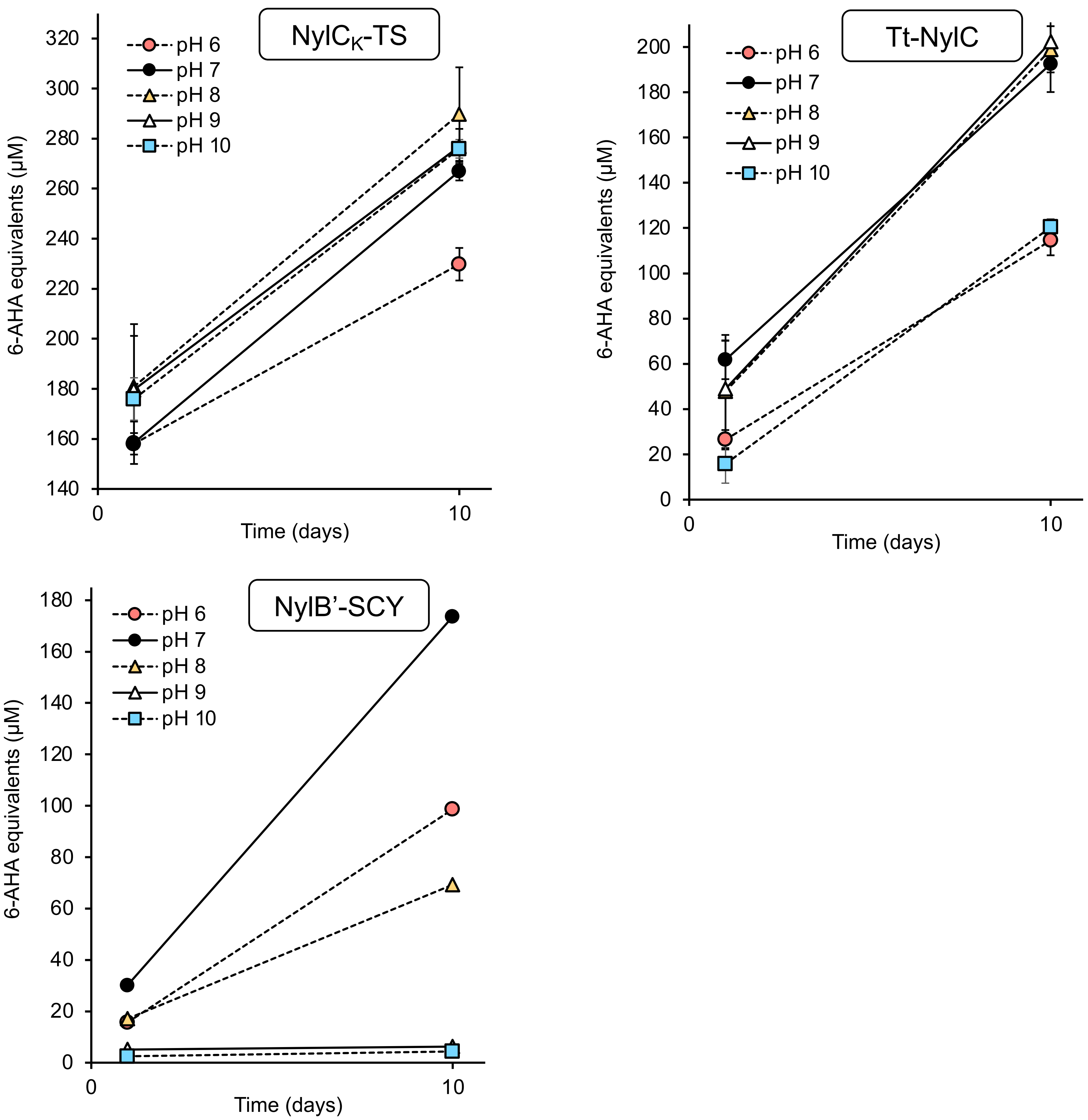

Supplementary Figure 22. Reaction profiles of enzymatic PA6 deconstruction with different enzymes at different pHs. Total released linear 6-AHA oligomers, represented as 6-AHA monomer equivalents, in reactions with 1 µM NylC$_K$-TS (60 °C), Tt-NylC (60 °C) or NylB'-SCY (50 °C) and 13 mg PA6 (0.08 mM enzyme/g PA6 film, 0.65 wt% substrate loading) over the course of 10 days, in reaction buffers from pH 6-10 (100 mM buffer, pH 6: citrate, pH7/8: NaPi, pH 9/10 Gly-OH, 150 mM NaCl) at 60 °C. Reactions were carried out in triplicate (n=3); error bars represent the standard deviation of the replicate measurements; circle, square, and triangle points represent the mean value of the triplicate measurements; the error bar centers are the means of the replicate measurements.

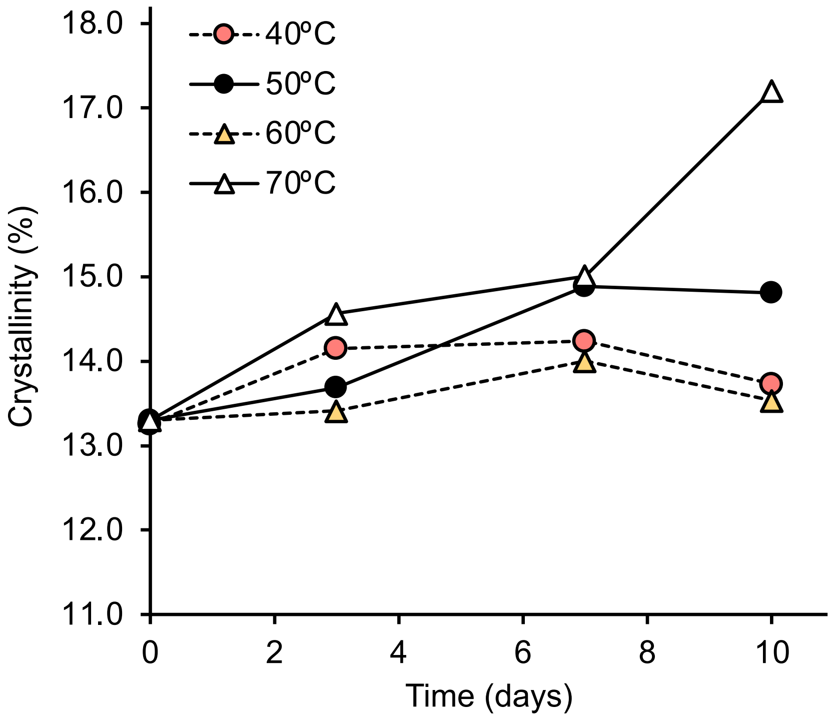

Supplementary Figure 23. Crystallinity changes in no enzyme control reactions over time at different temperatures. Crystallinity changes of PA6 film were determined by DSC over the course of 10 days. For each temperature from 40-70 °C, 13 mg PA6 film (0.65 wt% substrate loading) was incubated in reaction buffer (100 mM NaPi buffer, pH 7.5, 150 mM NaCl). Points represent single measurements (n=1).

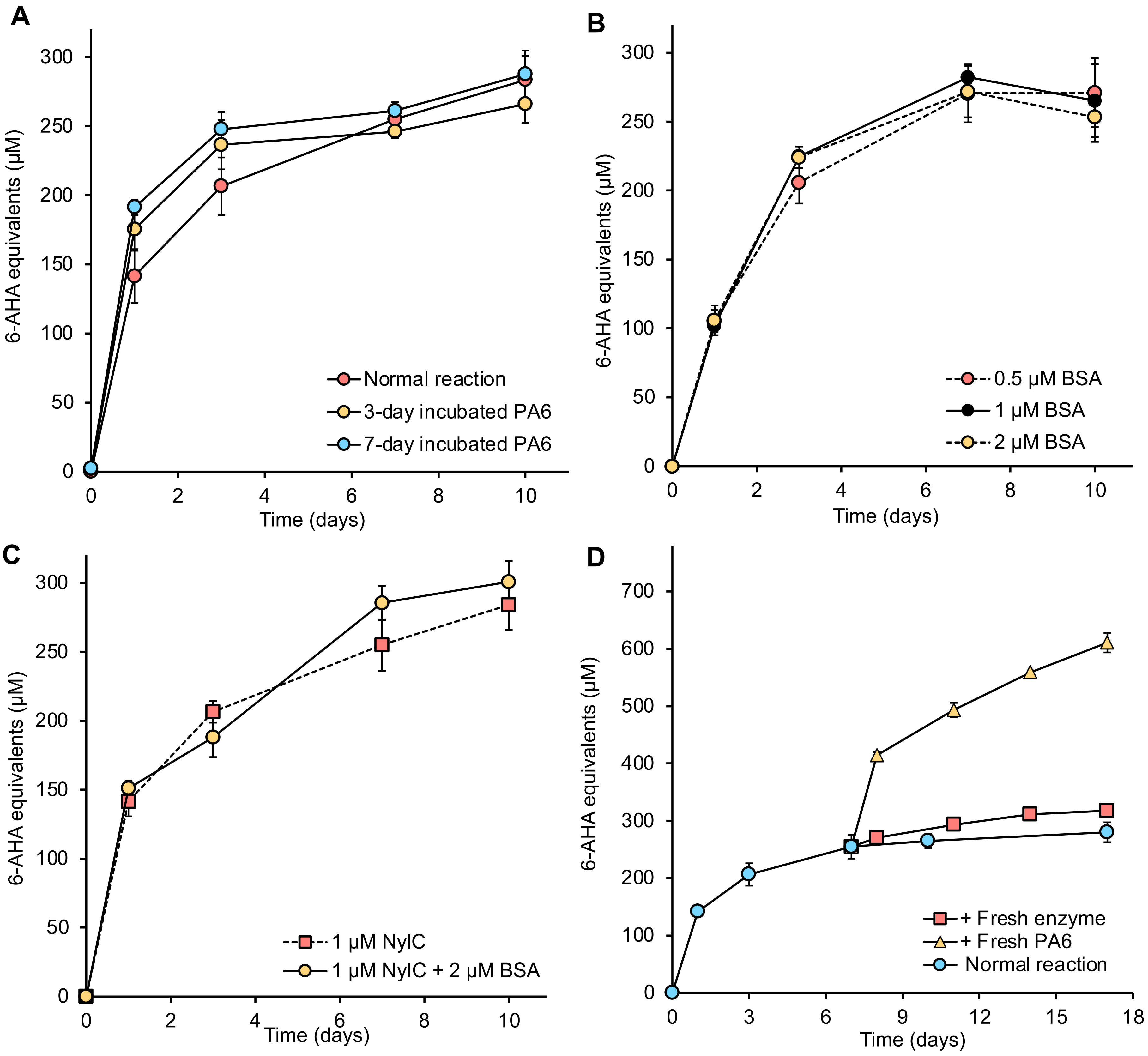

Supplementary Figure 24. Reaction profiles of NylC$_K$-TS PA6 deconstruction reactions to understand reaction plateau. **A.** Total released linear 6-AHA oligomers, represented as 6-AHA monomer equivalents, in reactions with 1 µM NylC$_K$-TS and 13 mg PA6 (0.08 mM enzyme/g PA6 film, 0.65 wt% substrate loading) over the course of 10 days. PA6 film squares were incubated in reaction buffer for either 0 days (Normal reaction), 3 days or 7 days prior to enzyme addition. **B.** Total released linear 6-AHA oligomers in reactions with 0.01 µM NylC$_K$-TS and 13 mg PA6 (0.8 µM enzyme/g PA6 film, 0.65 wt% substrate loading) over the course of 10 days, supplemented with 0.5-2.0 µM BSA. **C.** Total released linear 6-AHA oligomers in reactions with 1 µM NylC$_K$-TS and 13 mg PA6 (0.08 mM enzyme/g PA6 film, 0.65 wt% substrate loading) over the course of 10 days, supplemented with 2 µM bovine serum albumin (BSA). **D.** Total released linear 6-AHA oligomers in reactions started with 1 µM NylC$_K$-TS and 13 mg PA6 (0.08 mM enzyme/g PA6 film, 0.65 wt% substrate loading, normal reaction), where following reaction for 7 days, either fresh enzyme (1µM) or fresh PA6 film (13 mg) was added, and the reaction allowed to progress. For A, B, C, and D reactions were conducted in 100 mM NaPi buffer, pH 7.5, 150 mM NaCl at 60 °C; reactions were carried out in triplicate (n=3); error bars represent the standard deviation of the replicate measurements; circle, square and triangle points represent the mean value of the triplicate measurements; the error bar centers are the means of the replicate measurements.

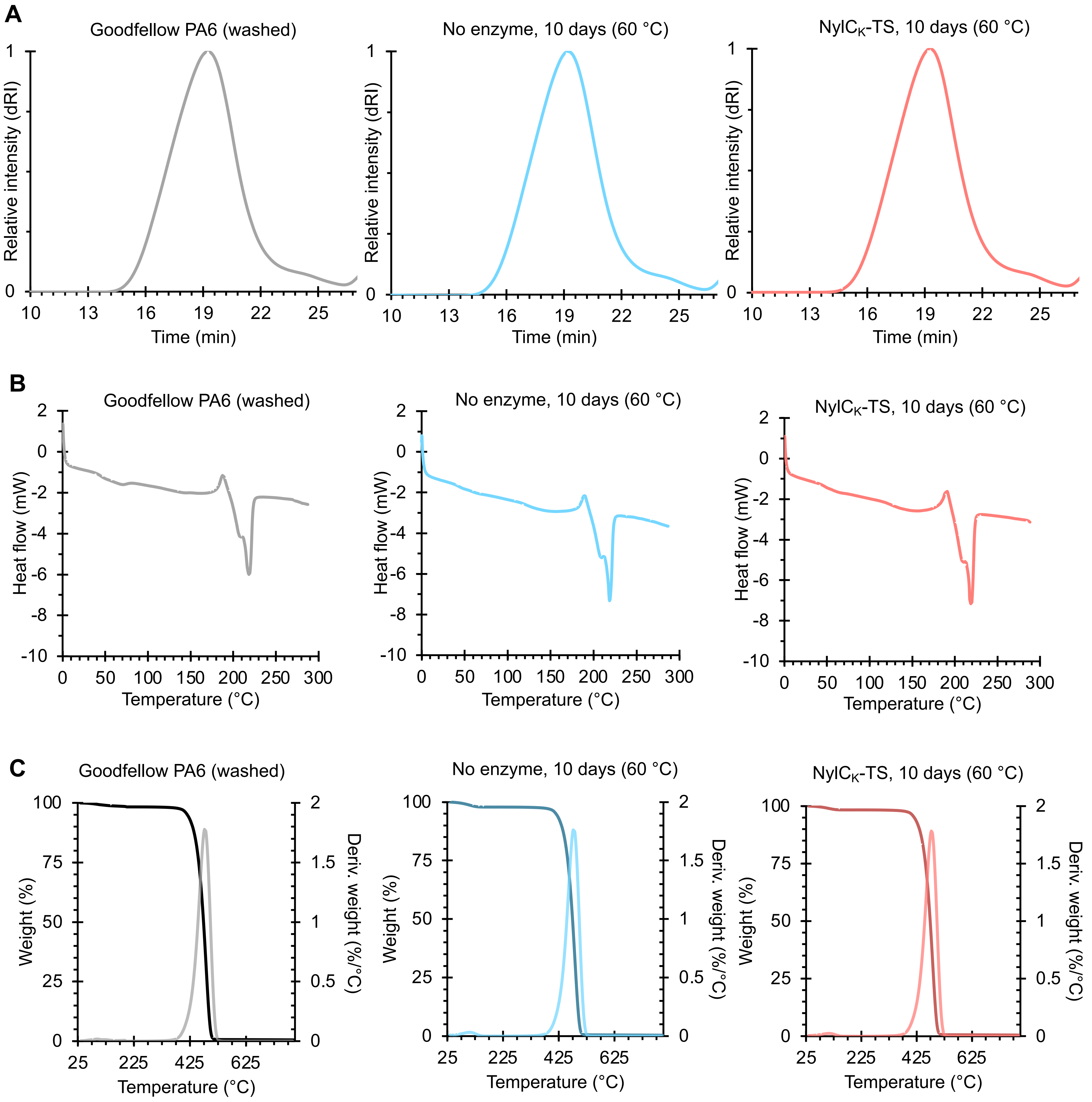

Supplementary Figure 25. Substrate characterization of PA6 films incubated with and without enzyme. Comparison of **A.** GPC, measured with the differential refractive index (dRI) detector **B.** DSC, and **C.** TGA plots for reactions with or without enzyme after 10 days of incubation at 60 °C versus the washed PA6 film prior to reaction. No enzyme reactions contained 13 mg PA6 (0.65 wt% substrate loading) in reaction buffer (100 mM NaPi buffer, pH 7.5, 150 mM NaCl). $NylC_K$-TS reactions contained 1 µM $NylC_K$-TS and 13 mg PA6 (0.08 mM enzyme/g PA6 film, 0.65 wt% substrate loading) in reaction buffer (100 mM NaPi buffer, pH 7.5, 150 mM NaCl). For the TGA plots, the darker colored lines represent the weight (%) (left y-axis), and the lighter lines the derivative weight (%) (right y-axis). Plots represent single measurements (n=1).

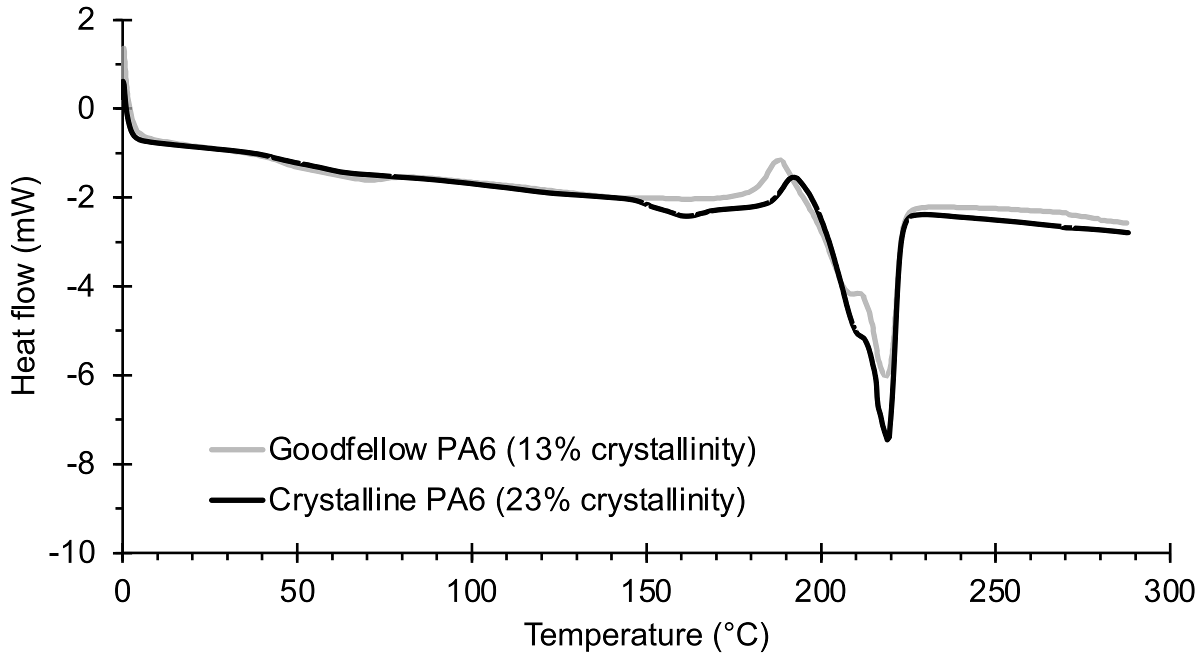

Supplementary Figure 26. DSC traces for Goodfellow PA6 film and crystalline PA6 film. DSC plots of the in-house prepared crystalline PA6 film (23.0% crystallinity) versus the Goodfellow PA6 film (13.2% crystallinity) used in enzyme reactions. For DSC, the PA6 was dried at 40 °C for 24 h prior to analysis to remove any residual water. The DSC analysis presented here was conducted on the unmodified samples prior to washing or reaction. Plots represent single measurements (n=1).

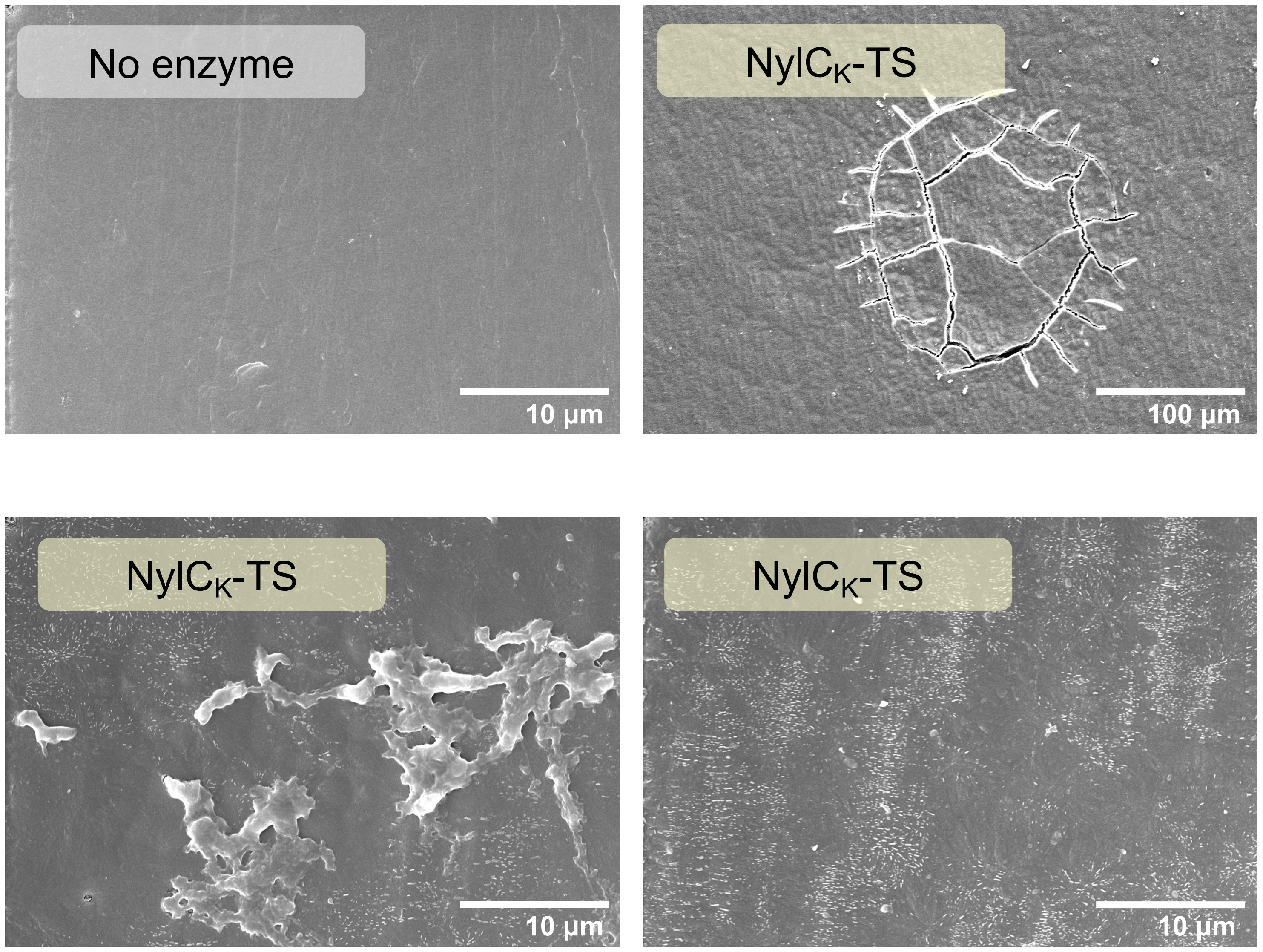

Supplementary Figure 27. SEM images of PA6 films incubated with $NylC_K$-TS. Example SEM images of PA6 films incubated in reaction buffer (100 mM NaPi buffer, pH 7.5, 150 mM NaCl) at 60 °C for 10 days, either without enzyme (no enzyme) or with 1 µM $NylC_K$-TS and 13 mg PA6 (0.08 mM enzyme/g PA6 film, 0.65 wt% substrate loading). SEM images show additional interesting features of the PA6 film following $NylC_K$-TS depolymerizations. Ten images were collected for each experiment, with those shown being representative of the set. Scale bar either 10 or 100 µm as detailed in the image.

Supplementary Table 3. Materials characterization of alternative PA6 substrates. Summary of DSC, GPC and TGA data collected for alternative PA6 substrates. The molar mass dispersity index is represented by *Đ*. For the sample preparation, "unmodified" samples were analyzed as received from the supplier or as originally synthesized, apart from DSC measurements, where the PA6 was dried in a vacuum oven at 40 °C for 24 h prior to analysis to remove any residual water, "washed" samples were analyzed following the washing protocol described in the Methods prior to reaction, "no enzyme" samples were incubated in reaction buffer (100 mM NaPi buffer, 150 mM NaCl, pH 7.5) for the described amount of time at 60 °C in the absence of enzyme, and "+NylC$_K$-TS" samples were analyzed following enzymatic reactions for the described amount of time at 60 °C (1 µM of enzyme, 0.08 mM enzyme/g PA6 film, 0.65% wt PA6 substrate loading, 100 mM pH 7.5 NaPi buffer with 150 mM NaCl). Dashes indicate where data could not be collected. The mass loss measured during TGA analysis can be attributed to water loss as it occurred at around 100 °C. Goodfellow PA6 powder (5-50 µM particle size) is referred to GF PA6 powder; Goodfellow PA6 film (0.5 mm thickness) is referred to as "GF thick PA6 film". Values represent a single measurement (n=1).

Substrate	Sample preparation	Time incubated (days)	T_g (°C)	% Crystallinity	M_n (kDa)	M_w (kDa)	*Đ*	$T_{D,50}$ (°C)	Mass loss (wt%)
GF PA6 powder	Unmodified	0	-	47.6	14.4	33.0	2.3	454.2	1.1
	Washed	0	-	47.7	11.9	31.9	2.7	459.9	0.4
GF PA6 thick film	Unmodified	0	59.3	16.5	27.6	47.8	1.7	475.8	1.4
	Washed	0	53.3	16.9	28.4	47.7	1.7	475.2	1.8
GF PA6 powder	No enzyme	10	-	25.6	13.5	31.4	2.3	470.9	0.2
	+ NylC$_K$-TS	10	-	37.2	13.4	32.8	2.4	472.8	0.6
GF PA6 thick film	No enzyme	10	49.5	20.7	28.9	42.5	1.5	474.5	1.3
	+ NylC$_K$-TS	10	56.2	21.5	31.6	46.3	1.5	475.3	1.5

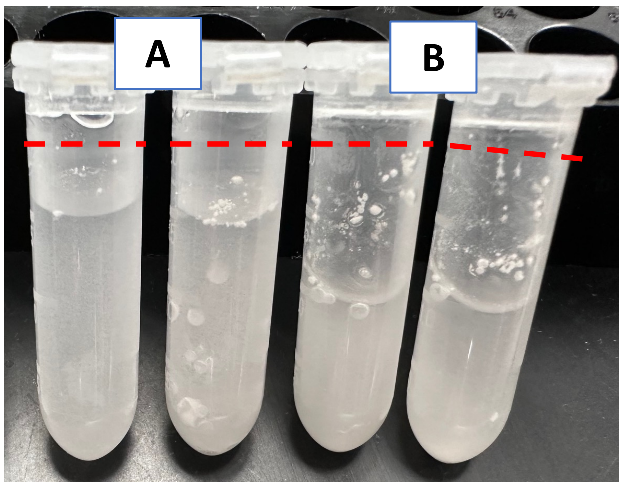

Supplementary Figure 28. Change in reaction volume of reactions with PA6 powder. Example image showing the changes in reaction volume following reactions with **A.** PA6 powder (13mg, 47.6% crystallinity, particle size: 5-50µm, sourced from Goodfellow) without enzyme addition, incubated at 60 °C over the course of 7 days, and **B.** PA6 powder (13mg, 47.6% crystallinity, particle size: 5-50µm) incubated with 2 µM NylC$_K$-TS at 60 °C over the course of 7 days. Reactions were carried out in duplicate (n=2) and started with 2 mL liquid volume, which is marked on the tubes as a red dotted line.

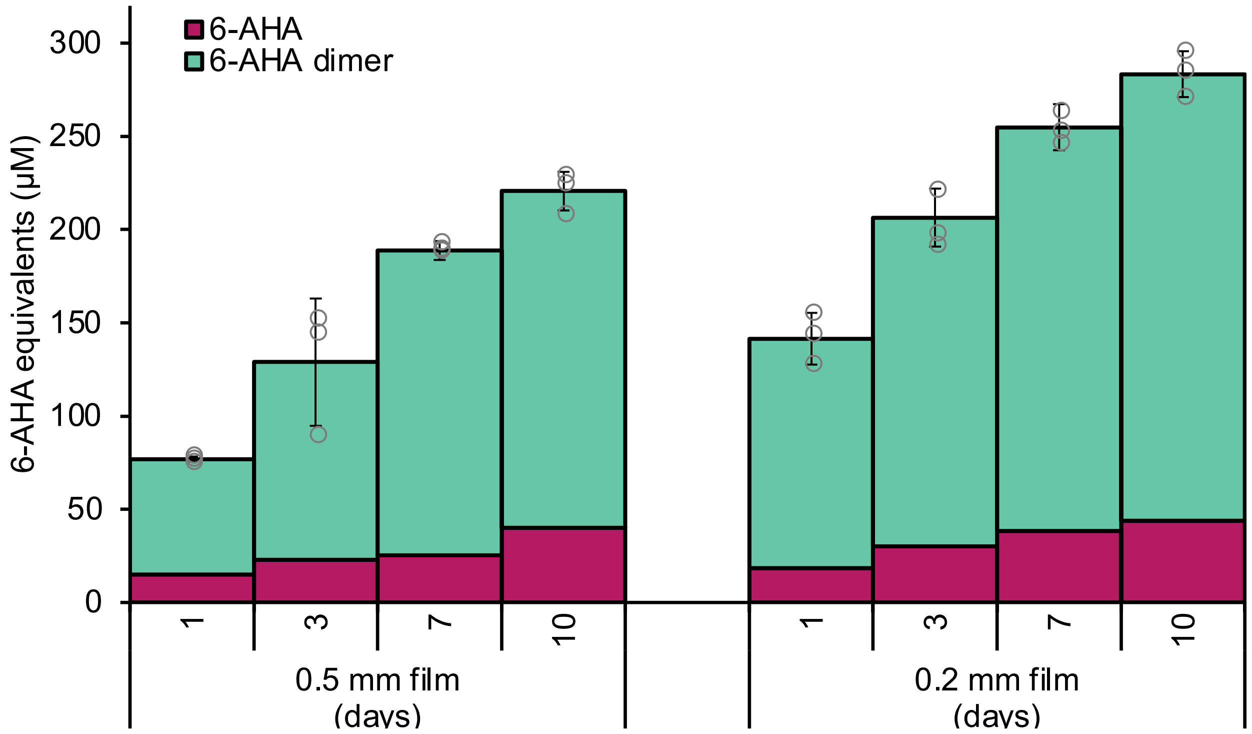

Supplementary Figure 29. Reactions of NylC$_K$-TS with different thicknesses of PA6 film. Total released linear 6-AHA oligomers with NylC$_K$-TS, following reaction with 13 mg PA6 film of either 0.5 mm thickness (two 0.35 cm x 0.35 cm squares) or 0.2 mm thickness (two 0.5 cm x 0.5 cm squares). Reactions contained 1 µM enzyme (0.08 mM enzyme/g PA6 film, 0.65 wt% substrate loading) and were incubated at 60 °C over the course of 10 days in reaction buffer (100 mM NaPi buffer, pH 7.5, 150 mM NaCl). Reactions were carried out in triplicate (n=3), error bars show the standard deviation of the replicate measurements, the error bar centers are the means of the replicate measurements, and the replicate measurements are represented as grey circles.

Supplementary Table 4. Summary of GPC data for three different batches of Goodfellow PA6. For further substrate characterization analyses in the study, only batch, unmodified (1), was used. Values represent a single measurement (n=1).

Substrate	Sample preparation (batch)	M_n (kDa)	M_w (kDa)	*Đ*
Goodfellow PA6 film	Unmodified (1)	33.8	50.4	1.5
	Unmodified (2)	33.9	50.4	1.5
	Unmodified (3)	32.8	51.5	1.5

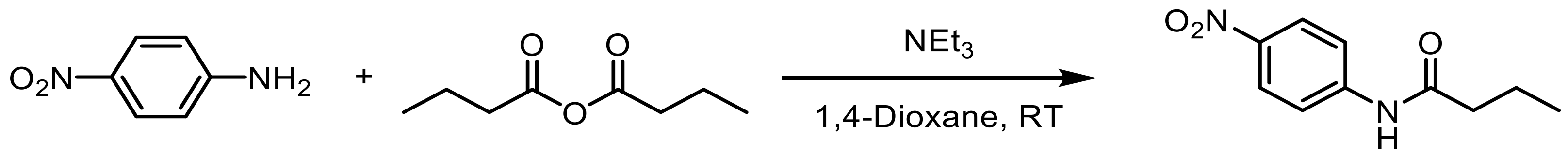

Supplementary Figure 30. Chemical synthesis of *N*-(4-nitrophenyl)butanamide (N4NB). Adapted from a literature procedure.[1] 4-nitroaniline (0.50 g, 3.62 mmol, 1 equiv.) was dissolved in anhydrous dioxane (0.6 mL) and cooled to 0 °C. Triethylamine (0.7 mL, 5.03 mmol, 1.4 equiv.) was added dropwise, and then allowed to warm to RT. Butyric anhydride (1.43 g, 9.05 mmol, 2.5 equiv.) in dioxane (0.6 mL), was added dropwise to the reaction, and stirred at RT for 16 h. A saturated aqueous solution of $NaHCO_3$ (40 mL) was added, and the product was extracted with EtOAc (2 x 20 mL). The combined organic layers were washed with brine and dried over anhydrous Na_2SO_4, filtered, and concentrated to obtain a brown oily residue. The product was precipitated from hexane as a brown solid (0.42 g, 2.10 mmol, 57% yield). ^{1}H NMR (DMSO-d_6, 400 MHz) δ 10.49 (s, 1H), 8.21 (d, J = 9.37 Hz, 2H), 7.48 (d, J = 9.37 Hz, 2H), 2.36 (t, J = 7.36 Hz, 2H), 1.62 (hex, J = 7.36 Hz, 2H), 0.92 (t, J = 7.36 Hz, 3H) ppm (^{1}H NMR spectrum available in **Supplementary Figure 31**).

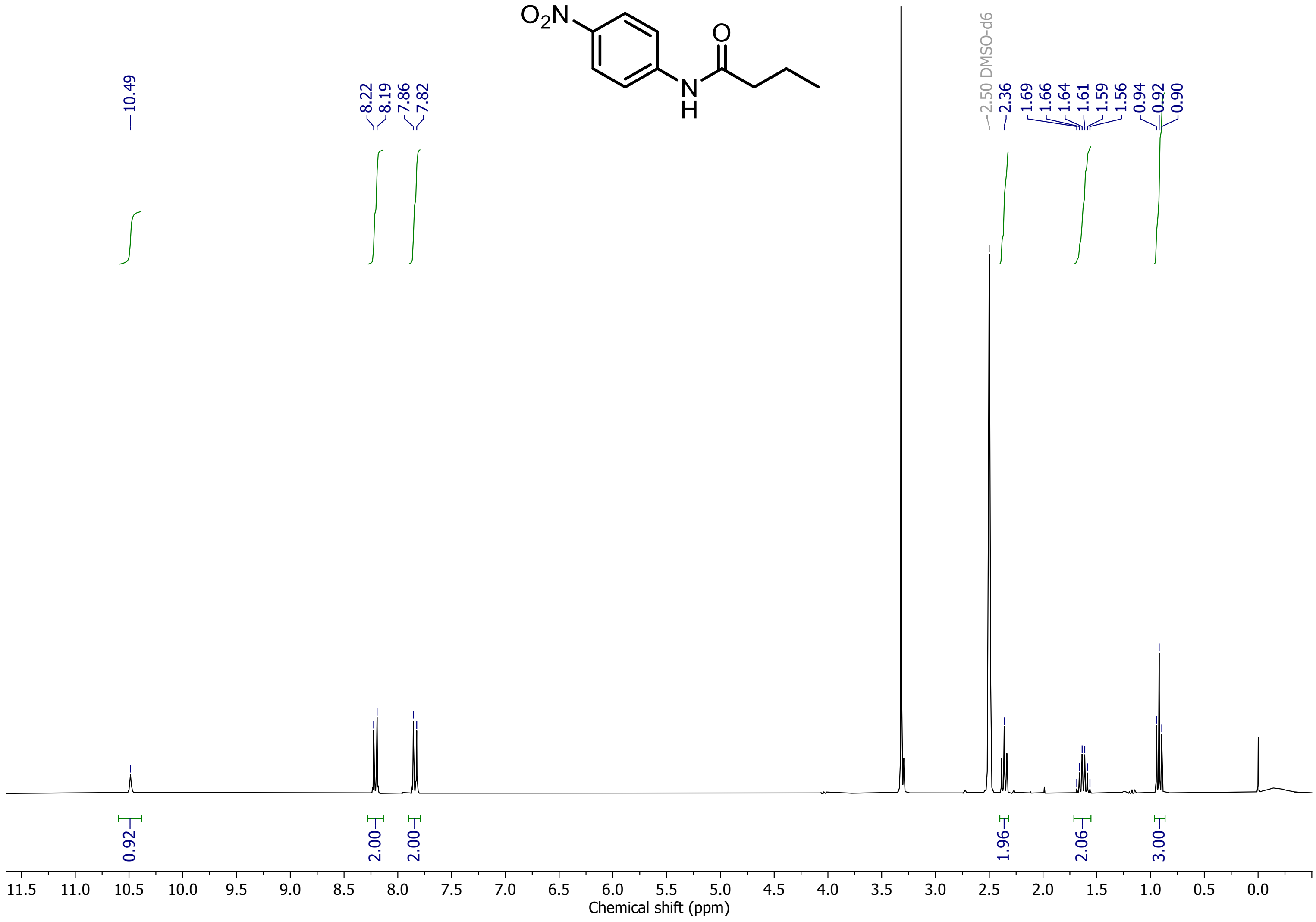

Supplementary Figure 31. ^{1}H NMR spectrum for *N*-(4-nitrophenyl)butanamide (NB4N). ^{1}H NMR (DMSO-d_6, 400 MHz) δ 10.49 (s, 1H), 8.21 (d, J = 9.37 Hz, 2H), 7.48 (d, J = 9.37 Hz, 2H), 2.36 (t, J = 7.36 Hz, 2H), 1.62 (hex, J = 7.36 Hz, 2H), 0.92 (t, J = 7.36 Hz, 3H) ppm.

Supplementary Table 5. PA6 deconstruction product dMRM transitions. Summary of optimized qualifying and quantifying mass to charge ratios (m/z), dMRM transitions and accompanying collision energies (CE), and fragmentor voltages (V).

Analyte name	Analyte formula	Precursor ion (*m/z*)	Ion	MRM quantifying transition (*m/z*)	CE (V)	Fragmentor (V)	MRM qualifying transition (*m/z*)	CE (V)
ε-caprolactam	$C_6H_{11}NO$	114.1	$[M-H]^+$	114.1 → 55.2	28	107	114.1 → 96.1	16
6-AHA	$C_6H_{13}NO_2$	132.1	$[M-H]^+$	132.1 → 114.1	8	65	132.1 → 79.1	16
6-AHA cyclic-dimer	$C_{12}H_{22}N_2O_2$	227.2	$[M-H]^+$	227.2 → 96.1	24	134	227.2 → 114.1	24
6-AHA dimer	$C_{12}H_{24}N_2O_3$	245.2	$[M-H]^+$	245.2 → 114.1	24	87	245.2 → 228.1	16
6-AHA cyclic-trimer	$C_{18}H_{33}N_3O_3$	340.3	$[M-H]^+$	340.3 → 114.1	36	N/A	N/A	N/A
6-AHA trimer	$C_{18}H_{35}N_3O_4$	358.3	$[M-H]^+$	358.3 → 114.1	36	124	358.3 → 245.1	24
6-AHA tetramer	$C_{24}H_{46}N_4O_5$	471.4	$[M-H]^+$	471.4 → 114.1	48	N/A	N/A	N/A
6-AHA pentamer	$C_{30}H_{57}N_5O_6$	584.4	$[M-H]^+$	584.4 → 114.1	72	N/A	N/A	N/A

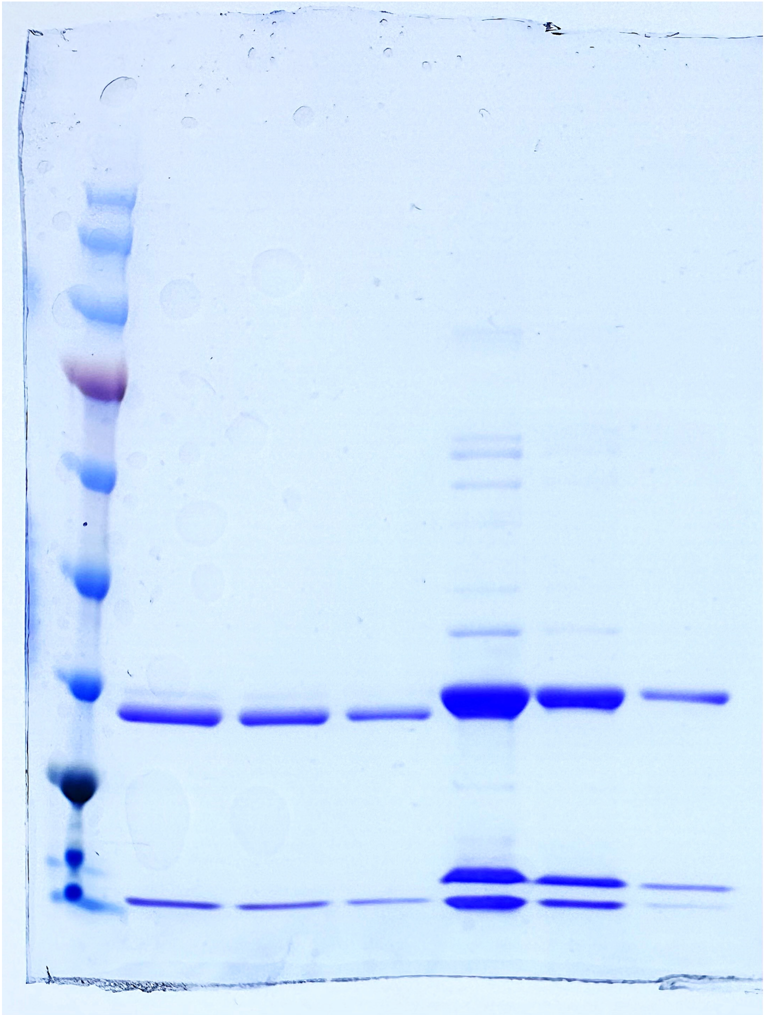

Supplementary Figure 32. Uncropped SDS gel image from Supplementary Figure 6.

Supplementary References

1. Conole, D. *et al.* Synthesis and methemoglobinemia-inducing properties of benzocaine isosteres designed as humane rodenticides. *Bioorg. Med. Chem.* **22**, 2220–2235 (2014).
