# Supplementary material for: Natural diversity screening, assay development, and characterization of nylon-6 enzymatic depolymerization
Source: Nat Commun. 2024 Feb 9;15:1217. doi: 10.1038/s41467-024-45523-5 (PMC10858056; doi:10.1038/s41467-024-45523-5)
Supplement: Supplementary file 3 — Description of Additional Supplementary files [file 41467_2024_45523_MOESM3_ESM.pdf]

Description of Additional Supplementary files

File name: Supplementary Data 1
Description: Panel of enzymes selected for their potential PA6 deconstruction activity. The table includes each enzyme's purported source, groupings used in the study (Group) and reasons for selection, where PA=polyamide, PET= poly(ethylene) terephthalate, PUR= polyurethane, UR=urethane, AL= Academic literature and P=Patent. The yield of protein was determined from a 100 mL E. coli expression culture purified by Ni-Nta affinity chromatography as described in the Methods. Five enzymes were from commercial sources (yield=C); one enzyme could not be expressed (no yield). References are detailed below the table, those obtained via homology searches have no applicable reference (N/A).

File name: Supplementary Data 2
Description: Details of enzymes used in the study. Summary table of enzymes used in the study including, enzyme classification (EC number), gene expression vector, amino acid (AA) sequence, and extinction coefficient used. All plasmid constructs have been deposited at AddGene (https://www.addgene.org/Gregg_Beckham/).
